# Ascorbic Acid Potentiates Cisplatin-Induced Antitumor Activity in HeLa Cervical Cancer Cells: Oxidative Stress, Mitochondrial Dysfunction, Apoptosis, and 3D Spheroid Responses

**DOI:** 10.3390/ijms27219526

**Published:** 2026-10-25

**Authors:** Mehmet Emin Ayağ, Harun Egemen Tolunay, Mehmet Cudi Tuncer, Şamil Öztürk

**Affiliations:** 1 Department of Gynecology and Obstetrics, Mehmet Emin Ayağ Practice, Mardin 47000, Turkey; 2 Department of Gynecology and Obstetric, Dr. Harun Egemen Tolunay Practice, Yasam Caddesi, AkPlaza, No: 7, Apartment: 19, Ankara 06560, Turkey; dregementolunay@hotmail.com; 3 Department of Anatomy, Faculty of Medicine, Dicle University, Diyarbakır 21280, Turkey; 4 Vocational School of Health Services, Çanakkale Onsekiz Mart University, Çanakkale 17100, Turkey; ozturksamil@outlook.com

**Keywords:** ascorbic acid, cisplatin, cervical cancer, combination index, ROS, oxidative stress

## Abstract

Cisplatin is widely used in cervical cancer chemotherapy; however, dose-limiting toxicity and the development of treatment resistance restrict its therapeutic efficacy. Ascorbic acid (AsA) may exert pro-oxidant and anticancer effects at pharmacological concentrations and has the potential to enhance the activity of conventional chemotherapeutic agents. This study aimed to investigate whether AsA enhances the anticancer activity of CDDP in HeLa cervical cancer cells and to characterize the underlying mechanisms, with particular emphasis on oxidative stress, mitochondrial dysfunction, apoptotic signaling, and the persistence of the combination effect in a three-dimensional (3D) tumor spheroid model. The effects of AsA and CDDP, alone and in combination, were evaluated in HeLa cells using cell viability and Chou–Talalay combination index (CI) analyses. Apoptosis, cell-cycle distribution, intracellular reactive oxygen species (ROS), N-acetylcysteine (NAC)-mediated antioxidant rescue, mitochondrial membrane potential (ΔΨm) using JC-1 (5,5′,6,6′-tetrachloro-1,1′,3,3′-tetraethylbenzimidazolylcarbocyanine iodide), caspase-3/7 activity, and apoptosis-related gene expression were assessed. The antitumor activity of the combination was further evaluated in 3D HeLa spheroids by assessing spheroid morphology, diameter, adenosine triphosphate (ATP)-based viability, and Live/Dead fluorescence staining. AsA and CDDP exhibited 48-h half-maximal inhibitory concentration (IC_50_) values of 3.73 mM and 3.93 µM, respectively, and their combination produced synergistic cytotoxicity (CI = 0.72). Total apoptosis increased from 6.8% in control cells to 47.6% following combined treatment, accompanied by increased ROS generation, caspase-3/7 activity, and G2/M accumulation. AsA + CDDP markedly decreased ΔΨm and increased the *BAX/BCL2* ratio and *TP53* expression, supporting activation of the mitochondrial apoptotic pathway. NAC attenuated ROS accumulation and partially rescued cell viability, supporting the involvement of oxidative stress in the combination response. In the 3D spheroid model, AsA + CDDP produced the greatest reduction in spheroid diameter and ATP-based viability, accompanied by marked structural disintegration and an increased relative EthD-1 red fluorescence-signal fraction. These findings identify AsA as a potential CDDP-sensitizing strategy and support further preclinical investigation. Because the study was limited to in vitro cell and spheroid models and did not establish in vivo efficacy or safety, future studies should validate the combination in more representative cervical cancer models and assess its efficacy and safety in vivo before clinical translation.

## 1. Introduction

Cancer remains a major global health challenge, with an estimated 20.6 million new cases and 9.8 million deaths worldwide in 2024 [1]. Cervical cancer remains an important health priority for women, and current global control efforts focus on HPV vaccination, screening, and treatment as the three pillars of cervical cancer elimination [2]. Although human papillomavirus (HPV) infection is the primary etiological factor for cervical cancer, tumor development and progression are driven by multiple molecular alterations, including disruption of cell-cycle control, evasion of apoptosis, oxidative stress, mitochondrial dysfunction, and genomic instability [3].

Cisplatin (CDDP) is a key chemotherapeutic agent widely used in the treatment of cervical cancer. CDDP forms covalent bonds with DNA, generating intra- and inter-strand cross-links that interfere with DNA replication and transcription and can trigger cell-cycle arrest and apoptosis [4,5]. However, the emergence of platinum resistance remains a major cause of treatment failure. CDDP-resistant cells may adapt through changes in glucose, lipid, and amino acid metabolism, as well as altered ferroptosis and autophagy; accordingly, combinations with cytotoxic, targeted, or immunotherapeutic agents are being investigated to improve treatment response [6,7]. Beyond resistance, the clinical use of CDDP is further constrained by dose-dependent toxicities, including nephrotoxicity, neurotoxicity, ototoxicity, and myelosuppression; susceptibility may also be influenced by pre-existing conditions and comorbidities [8,9]. Among these toxicities, renal injury is associated with transporter-mediated drug uptake, oxidative stress, mitochondrial dysfunction, DNA damage, inflammation, and regulated cell-death pathways. Although hydration remains the primary preventive measure, its protection may be limited, particularly in high-risk patients [8].

AsA is an essential water-soluble vitamin involved in cellular redox regulation and immune function. Beyond its antioxidant activity, vitamin C modulates innate and adaptive immune responses and supports T-cell development and differentiation through ten-eleven-translocation-mediated DNA demethylation [10]. At pharmacological concentrations, AsA can exert pro-oxidant and antitumor effects. In uterine serous carcinoma cell lines, high-dose ascorbate inhibited proliferation and invasion, induced cell-cycle arrest, cellular stress, DNA damage, and apoptosis; N-acetylcysteine attenuated some of these effects. Ascorbate also acted synergistically with carboplatin, enhancing growth inhibition, cellular stress, DNA damage, and caspase-3 activity [11]. A recent review further discusses the proposed anticancer mechanisms of AsA and its investigation in chemotherapy and other combination strategies [12]. The pharmacological use of AsA differs from nutritional oral intake because high-dose intravenous administration can achieve plasma concentrations that are not attainable orally; however, its clinical use remains under investigation, and potential risks include oxalate nephropathy and hemolysis in patients with glucose-6-phosphate dehydrogenase deficiency [13]. In a recent randomized trial in metastatic pancreatic cancer, intravenous pharmacological ascorbate was administered at 75 g three times weekly with gemcitabine and nab-paclitaxel, increasing serum ascorbate from micromolar to millimolar concentrations; the combination was associated with longer overall and progression-free survival without increased adverse-event frequency or severity [14]. These findings provide clinical context for pharmacological AsA use, while its effects with CDDP in cervical cancer require direct investigation.

Previous studies have directly examined AsA-mediated cisplatin sensitization in cervical cancer cells. In HeLa cells, vitamin C was reported to stabilize p53, increase BAX, decrease BCL2, and enhance sensitivity to cisplatin-induced cell-cycle arrest and apoptosis [15]. Subsequent work showed that pharmacological AsA increased ROS and enhanced HeLa-cell susceptibility to cisplatin; synergistic activity and redox- and p53-associated effects were also reported in SiHa cervical cancer cells [16,17]. Thus, the AsA–CDDP combination has prior experimental support, and the present study is intended to extend, rather than establish for the first time, this approach.

The combination of AsA and CDDP may enhance anticancer activity through distinct but interconnected mechanisms. CDDP-induced cellular damage combined with AsA-associated oxidative stress may increase the overall stress burden of tumor cells and facilitate activation of apoptotic pathways. Mitochondria represent an important convergence point for these processes because excessive ROS can disrupt ΔΨm, alter the balance between pro- and anti-apoptotic proteins, and promote downstream caspase activation. However, interactions between AsA and chemotherapeutic agents are not uniformly synergistic and may vary according to cell type, drug concentration, treatment sequence, and duration of exposure. Under certain experimental conditions, AsA has also been reported to attenuate CDDP-induced cytotoxicity [18]. Direct experimental characterization of the interaction is therefore necessary rather than assuming a potentiating effect.

Ascorbate-mediated reduction of platinum(IV) [Pt(IV)] compounds is a related but chemically distinct topic. Pt(IV) complexes can be reduced to Pt(II) species, but the reaction depends on the compound and its chemical environment. Kinetic studies of Pt(IV) anticancer prodrugs in human plasma showed that ascorbate can participate in their reduction, although several thiol reductants reacted more rapidly under the conditions tested; ascorbate therefore may not be the dominant reductant in plasma [19]. The present study evaluates AsA in combination with cisplatin, a Pt(II) agent; it does not test a Pt(IV) compound, compare the combination with satraplatin, or measure platinum speciation. Its findings should therefore not be interpreted as evidence of AsA-mediated Pt(IV) activation.

Oxidative stress is accompanied by multiple biochemical alterations, including lipid peroxidation, malondialdehyde (MDA) accumulation, glutathione (GSH) depletion, and changes in antioxidant enzyme activity. Simultaneous evaluation of ROS, MDA, GSH, and superoxide dismutase (SOD) can therefore provide complementary information regarding the redox effects of AsA and CDDP. Furthermore, antioxidant rescue using NAC provides a functional approach for determining whether increased oxidative stress contributes to treatment-induced cytotoxicity. Assessment of ΔΨm using JC-1 can further establish whether redox alterations are accompanied by mitochondrial dysfunction. Together with apoptosis-related markers such as *BAX*, *BCL2*, and *CASP3*, these analyses enable more comprehensive characterization of the relationship between oxidative stress, mitochondrial perturbation, and apoptotic cell death [20].

HeLa cells are a widely used experimental model of HPV-18-associated cervical cancer [21]. HaCaT cells, a spontaneously immortalized human keratinocyte cell line, can additionally provide a non-cancerous cellular comparator for evaluating differences in treatment response [22]. Nevertheless, conventional two-dimensional (2D) monolayer cultures cannot fully reproduce the cell–cell interactions, spatial organization, nutrient and oxygen gradients, and drug penetration characteristics of solid tumors. Three-dimensional tumor spheroids provide an intermediate model with greater tumor-like complexity and can therefore complement 2D experiments by determining whether treatment effects are retained under conditions that more closely reproduce the architecture of a solid tumor.

Bioinformatics approaches can further contribute to the interpretation of experimentally observed responses by identifying protein–protein interaction networks and biological pathways potentially associated with treatment effects. Functional enrichment and network analyses can integrate changes involving apoptosis, oxidative stress, proliferation, and cell-cycle regulation into a broader molecular framework and identify potential hub proteins involved in the cellular response to combination treatment [23,24].

Building on previous reports of AsA-mediated cisplatin sensitization in cervical cancer cells [15,16,17], this study evaluates the combination’s effects in both 2D HeLa cultures and 3D HeLa spheroids, with HaCaT cells included as a non-cancerous cellular comparator. We assessed drug interaction, apoptosis, cell-cycle distribution, ROS generation, NAC-mediated rescue, mitochondrial membrane potential, caspase-3/7 activity, and apoptosis-related gene expression, including *BAX*, *BCL2*, and *TP53*. Spheroid responses were evaluated using morphology, diameter, ATP-based viability, and Live/Dead fluorescence imaging, while bioinformatics analyses were used to explore associated protein–protein interaction networks and pathways. The study’s contribution is this integrated assessment of redox-associated mitochondrial and apoptotic responses across 2D and 3D models, extending earlier AsA–cisplatin studies rather than claiming to establish the combination for the first time.

## 2. Results

### 2.1. Concentration- and Time-Dependent Cytotoxic Effects of AsA and CDDP

**Figure 1:**
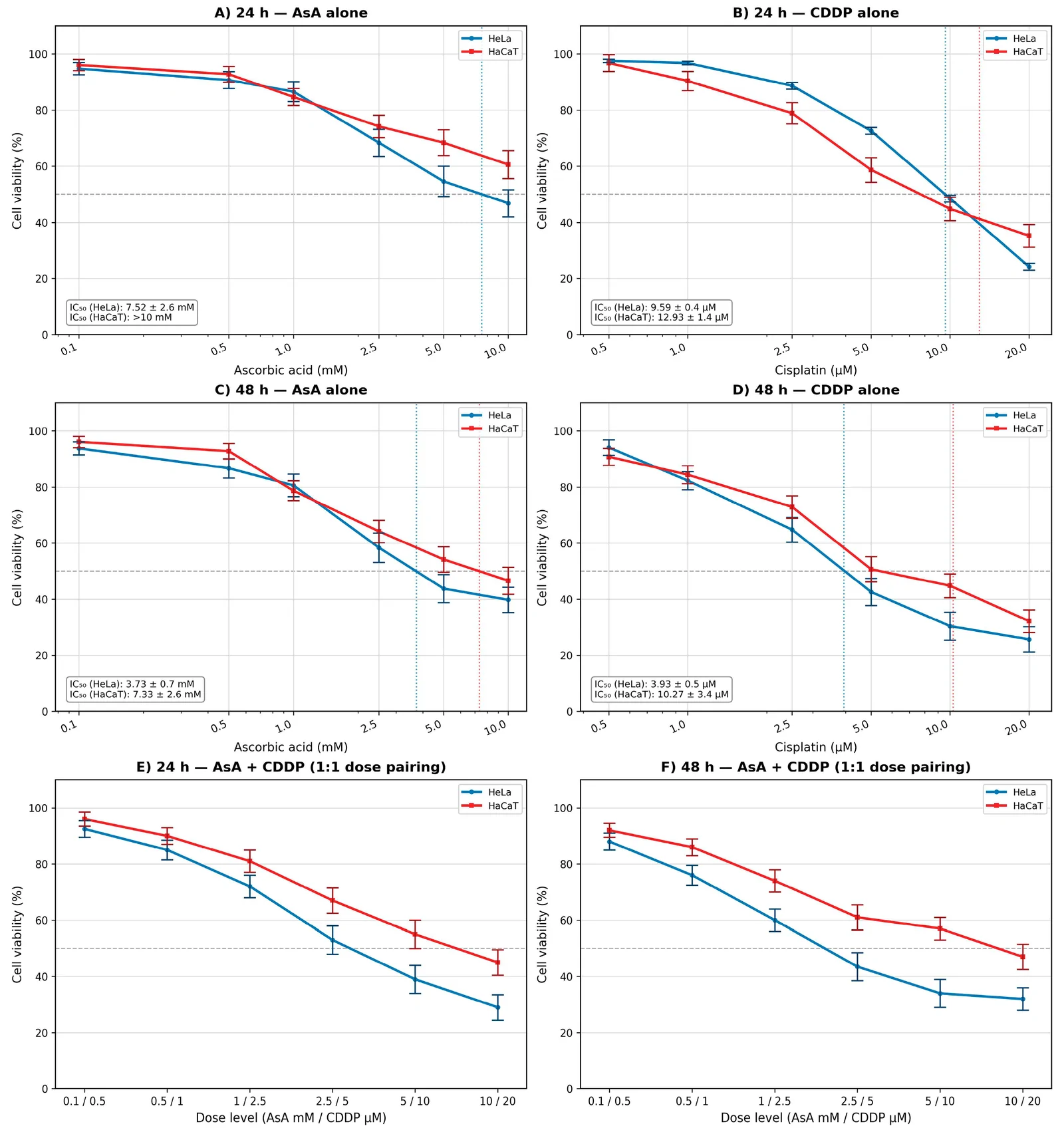
Concentration- and time-dependent effects of AsA, CDDP, and their paired-dose combination on the viability of HeLa and HaCaT cells. (**A**) Cell viability after 24 h of AsA treatment. (**B**) Cell viability after 24 h of CDDP treatment. (**C**) Cell viability after 48 h of AsA treatment. (**D**) Cell viability after 48 h of CDDP treatment. (**E**,**F**) Cell viability following combined AsA + CDDP treatment using paired doses for 24 h (**E**) and 48 h (**F**). Data are presented as mean ± SD from three independent biological experiments (*n* = 3). The horizontal dashed gray line indicates 50% cell viability. IC_50_ values shown in panels (**A**–**D**) are presented as mean ± SD from three independent experiments.

The XTT assay showed concentration-dependent reductions in HeLa and HaCaT cell viability after AsA or CDDP exposure (Figure 1A–D). In general, inhibition was greater after 48 h and in HeLa cells, although this pattern was not uniform across all treatments and doses. At 24 h, the IC_50_ values were 7.52 ± 2.6 mM for AsA and 9.59 ± 0.4 µM for CDDP in HeLa cells; the corresponding HaCaT values were >10 mM and 12.93 ± 1.4 µM. After 48 h, the IC_50_ values were 3.73 ± 0.7 mM for AsA and 3.93 ± 0.5 µM for CDDP in HeLa cells, and 7.33 ± 2.6 mM and 10.27 ± 3.4 µM, respectively, in HaCaT cells. The complete concentration–response profiles are shown in Figure 1.

Combined AsA + CDDP treatment produced a progressive reduction in cell viability with increasing paired doses in both cell lines (Figure 1E,F). After 24 h, viability at the highest tested combination (10 mM AsA + 20 µM CDDP) decreased to approximately 29% in HeLa cells and 45% in HaCaT cells (Figure 1E). After 48 h, the corresponding values were approximately 32% and 47%, respectively (Figure 1F). Thus, at the highest combination dose, viability was slightly higher after 48 h than after 24 h in both cell lines. This exception indicates that the combination response was not uniformly time-dependent at every dose. Across the combination dose range, HeLa cells generally exhibited lower viability than HaCaT cells, indicating greater cytotoxic responsiveness of HeLa cells to the combined treatment.

### 2.2. CI Analysis Reveals Differential AsA–CDDP Interactions in HeLa and HaCaT Cells

**Figure 2:**
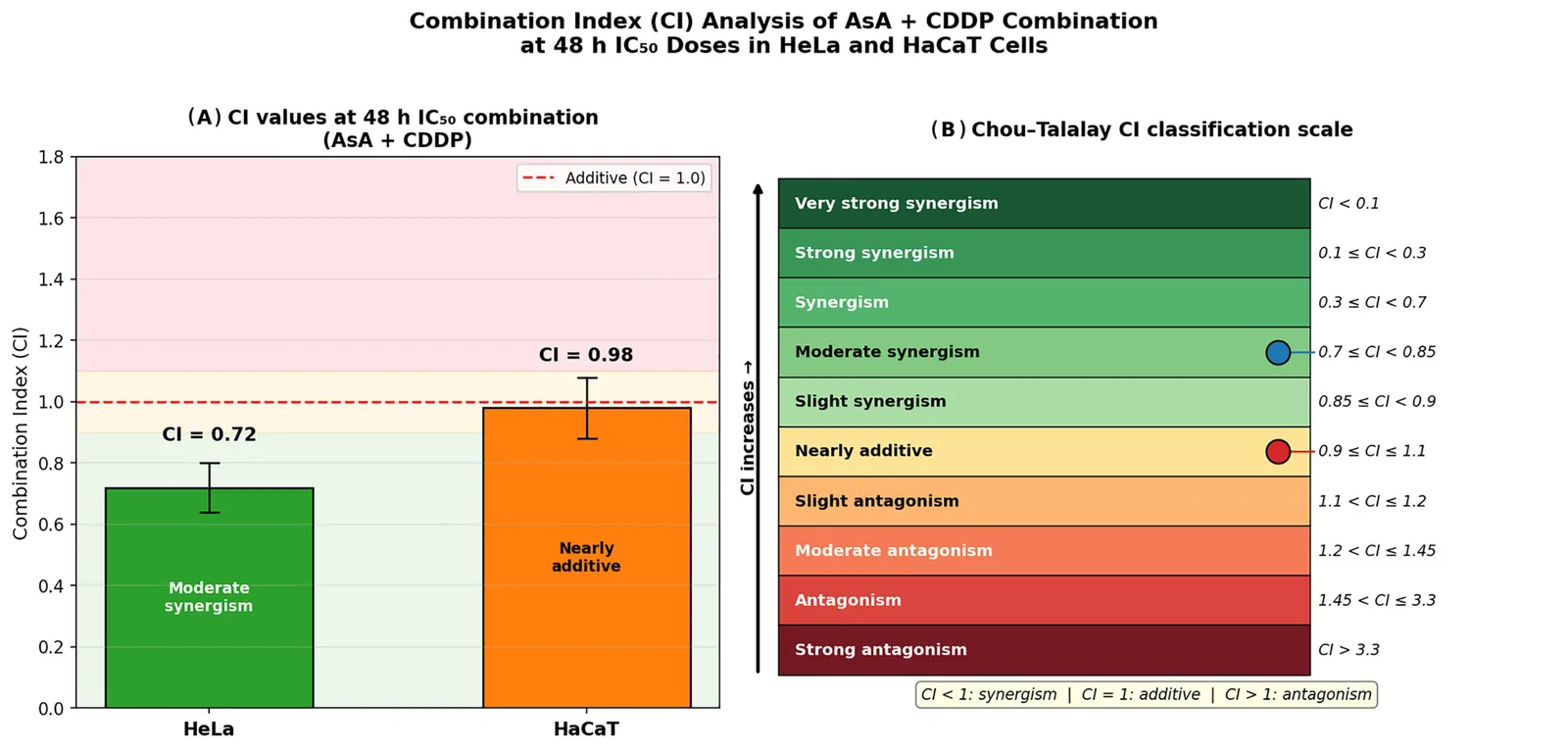
CI analysis of the AsA + CDDP combination in HeLa and HaCaT cells. Cells were treated with AsA and CDDP using their respective 48-h IC_50_-based concentrations, and drug interactions were evaluated using the Chou–Talalay CI method. (**A**) CI values for HeLa (CI = 0.72) and HaCaT (CI = 0.98) cells. Data are presented as mean ± SD (*n* = 3). The red dashed line indicates CI = 1.0. The shaded regions indicate synergistic (CI < 0.90), nearly additive (0.90 ≤ CI ≤ 1.10), and antagonistic (CI > 1.10) interactions. (**B**) Chou–Talalay CI classification scale showing moderate synergism for HeLa cells (CI = 0.72) and a nearly additive interaction for HaCaT cells (CI = 0.98), within the complete classification spectrum ranging from very strong synergism to strong antagonism.

The interaction between AsA and CDDP was quantitatively evaluated using the Chou–Talalay CI method. Under the 48-h IC_50_-based combination conditions, the CI value was 0.72 in HeLa cells and 0.98 in HaCaT cells (Figure 2A). Although both CI values were below the theoretical additive threshold of 1.0, their interaction classifications differed according to the predefined CI ranges shown in Figure 2B.

In HeLa cells, the CI value of 0.72 fell within the moderate synergism range (0.70 ≤ CI < 0.85), demonstrating a synergistic interaction between AsA and CDDP under the tested conditions. In contrast, the CI value of 0.98 obtained in HaCaT cells was within the nearly additive range (0.90 ≤ CI ≤ 1.10), indicating an interaction close to additivity rather than a comparable synergistic effect.

Overall, the CI analysis demonstrated a distinct interaction profile between the two cell lines, with moderate synergism in HeLa cells and a nearly additive interaction in HaCaT cells (Figure 2). This differential interaction profile was consistent with the lower cell viability observed in HeLa cells than in HaCaT cells across the AsA + CDDP paired-dose treatments (Figure 1E,F).

### 2.3. AsA and CDDP Induce Apoptosis-Associated Nuclear Morphological Alterations

**Figure 3:**
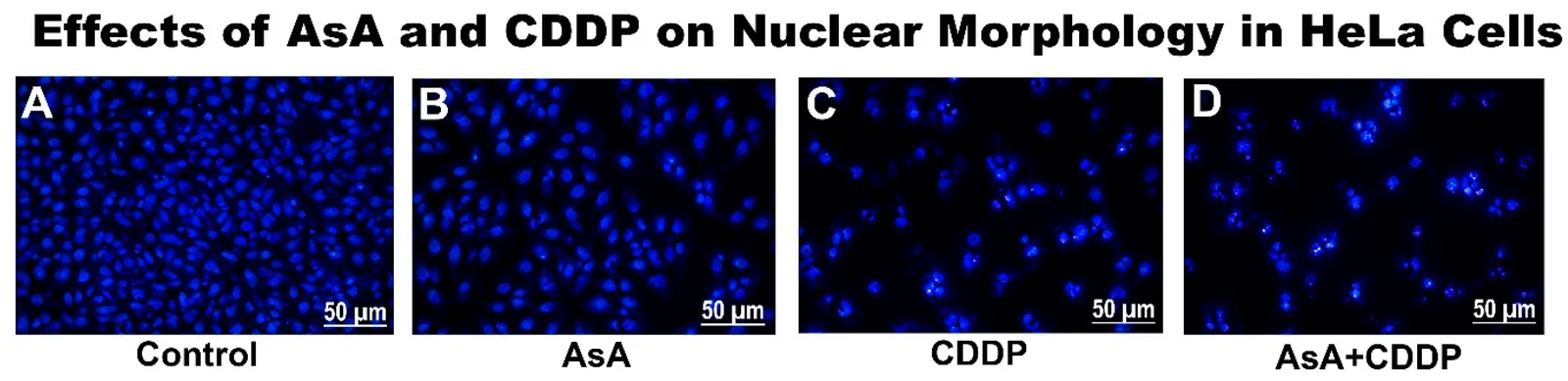
Representative fluorescence microscopy images of NucBlue (Hoechst 33342)-stained HeLa cells following treatment with AsA and CDDP. (**A**) Control, (**B**) AsA, (**C**) CDDP, and (**D**) AsA + CDDP. Representative images show treatment-associated differences in nuclear morphology and cellular distribution among the experimental groups. Magnification: ×10. Scale bar = 50 µm.

Nuclear morphological changes following AsA and CDDP treatment were evaluated in HeLa cells using NucBlue (Hoechst 33342) staining and fluorescence microscopy (Figure 3). Nuclear condensation was qualitatively identified by a smaller, more compact, and intensely Hoechst-positive nuclear profile, whereas nuclear fragmentation was identified by discrete Hoechst-positive bodies separated from the main nuclear profile. Control cells exhibited a high cell density with predominantly large, oval, and uniformly stained nuclei showing relatively homogeneous blue fluorescence (Figure 3A). Nuclear morphology was largely preserved, with minimal evidence of chromatin condensation or nuclear fragmentation.

Treatment with AsA at the 48-h IC_50_-based concentration resulted in a reduction in cell density accompanied by detectable alterations in nuclear morphology (Figure 3B). Compared with the control group, some nuclei exhibited increased fluorescence intensity and a more condensed appearance, although these changes were relatively limited.

More pronounced nuclear alterations were observed following CDDP treatment (Figure 3C), characterized by a marked reduction in cell density and an increased occurrence of small, intensely fluorescent, condensed, and irregular nuclear profiles. The AsA + CDDP combination produced the most prominent morphological alterations (Figure 3D), with markedly reduced cell density and numerous condensed and fragmented nuclear profiles exhibiting intense blue fluorescence. Overall, the progressive nuclear morphological changes observed across the treatment groups were consistent with enhanced apoptosis-associated nuclear damage, with the most pronounced alterations observed following combined AsA + CDDP treatment.

### 2.4. Semiquantitative Analysis Confirms Progressive Apoptosis-Associated Nuclear Damage

To further quantify the treatment-associated nuclear morphological alterations, nuclei were semiquantitatively scored on a 0–4 scale according to the degree of chromatin condensation and nuclear fragmentation. Score 0 represented morphologically normal nuclei (0–5% condensation), score 1 mild chromatin condensation (6–25%), score 2 moderate condensation with chromatin marginalization (26–50%), score 3 severe condensation with initial nuclear fragmentation (51–75%), and score 4 extensive nuclear fragmentation/karyorrhexis (>75%).

**Figure 4:**
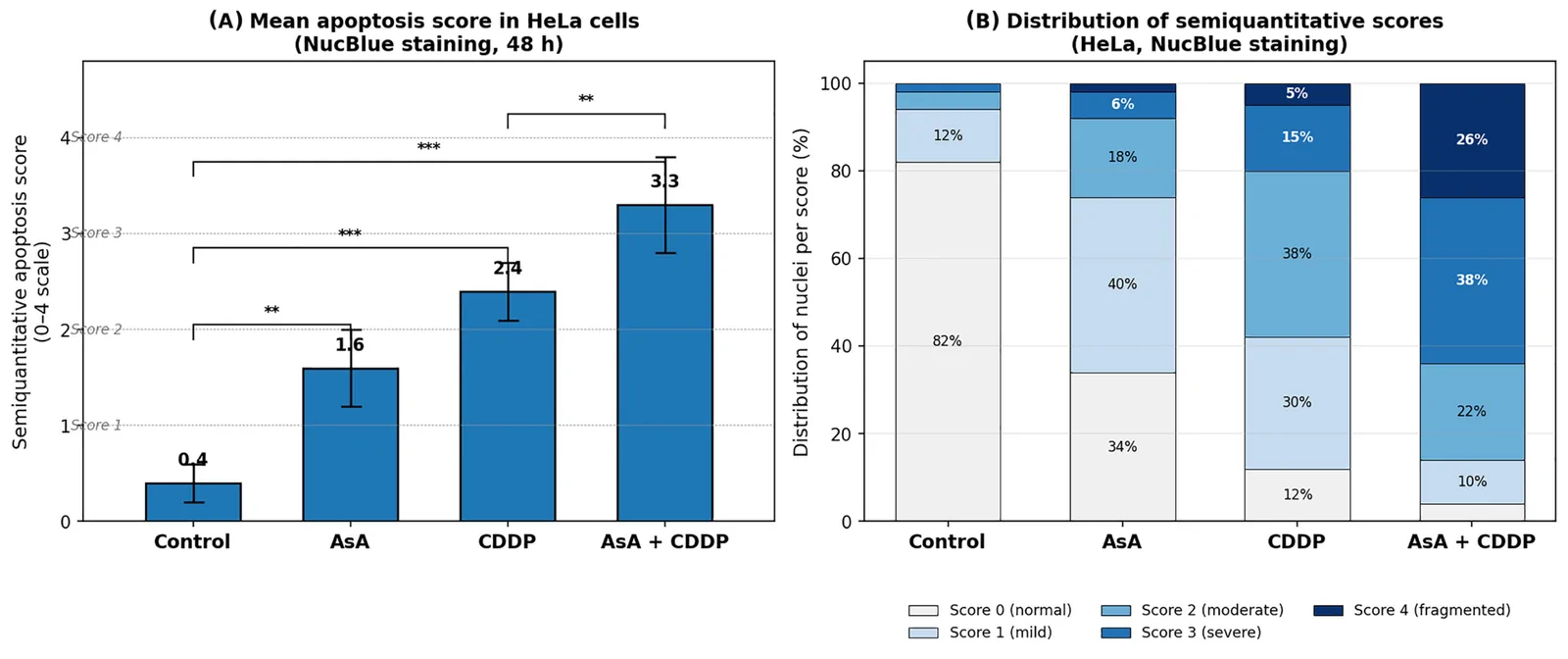
Semiquantitative analysis of apoptosis-associated nuclear morphological alterations in NucBlue-stained HeLa cells following AsA and CDDP treatment. HeLa cells were treated for 48 h with AsA, CDDP, or AsA + CDDP at the corresponding 48-h IC_50_-based concentrations and stained with NucBlue (Hoechst 33342). Nuclear morphology was semiquantitatively scored on a 0–4 scale according to the degree of chromatin condensation and nuclear fragmentation: score 0, normal; score 1, mild; score 2, moderate; score 3, severe; and score 4, fragmented. (**A**) Mean nuclear apoptosis score in each treatment group. (**B**) Percentage distribution of nuclei across the five morphological score categories. Data are presented as mean ± SD (*n* = 3). ** *p* < 0.01 and *** *p* **<** 0.001 for the comparisons indicated by brackets.

The mean nuclear apoptosis score was 0.4 ± 0.2 in control HeLa cells, with 82% of nuclei classified as score 0 (Figure 4A,B). AsA treatment significantly increased the mean score to 1.6 ± 0.4 (*p* < 0.01 vs. control), with 40% and 18% of nuclei classified as scores 1 and 2, respectively. CDDP produced a more pronounced increase in the mean score to 2.4 ± 0.3 (*p* < 0.001 vs. control), accompanied by a shift toward higher morphological scores, with 38% of nuclei classified as score 2 and 15% as score 3.

The AsA + CDDP combination resulted in the highest mean nuclear apoptosis score (3.3 ± 0.5), which was significantly higher than both the control (*p* < 0.001) and CDDP-alone (*p* < 0.01) groups (Figure 4A). Distribution analysis further showed that 38% of nuclei in the combination group were classified as score 3 and 26% as score 4, corresponding to a combined 64% of nuclei with severe or extensive nuclear alterations (Figure 4B). Overall, the score distribution progressively shifted from predominantly normal or mildly altered nuclei in the control and AsA groups toward higher scores following CDDP and particularly AsA + CDDP treatment, consistent with the nuclear morphological changes observed in Figure 3.

### 2.5. AsA Enhances CDDP-Induced Apoptosis in HeLa Cells

**Figure 5:**
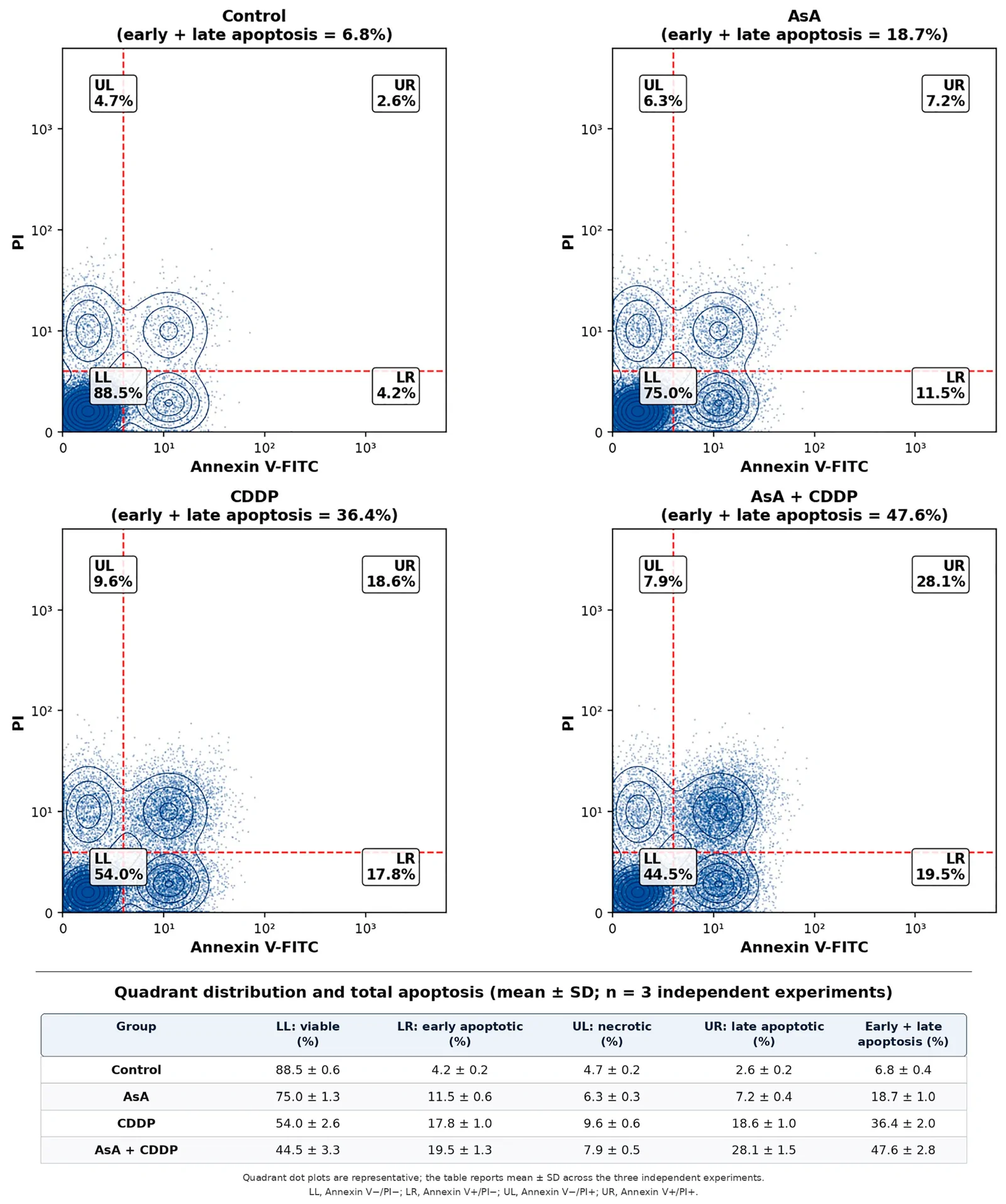
Annexin V-FITC/PI flow cytometric analysis of apoptosis in HeLa cells following 48-h treatment with AsA, CDDP, or their combination at the corresponding 48-h IC_50_-based concentrations. Quadrants indicate LL (Annexin V^−^/PI^−^, viable cells), LR (Annexin V^+^/PI^−^, early apoptotic cells), UR (Annexin V^+^/PI^+^, late apoptotic cells), and UL (Annexin V^−^/PI^+^, necrotic cells). The dot plots show representative results from three independent biological experiments. The summary values are presented as mean ± SD (*n* = 3). Total apoptosis was calculated as the sum of the early- and late-apoptotic populations (LR + UR): 6.8 ± 0.4% in the control group, 18.7 ± 1.0% in the AsA group, 36.4 ± 2.0% in the CDDP group, and 47.6 ± 2.8% in the AsA + CDDP group.The red dashed vertical and horizontal lines indicate the quadrant boundaries used to distinguish cell populations according to Annexin V-FITC and PI fluorescence intensities.

Annexin V-FITC/PI flow cytometric analysis demonstrated a progressive increase in apoptotic cell populations following AsA and CDDP treatment (Figure 5). Data are presented as mean ± SD from three independent biological experiments. In control cells, early and late apoptosis accounted for 4.2 ± 0.2% and 2.6 ± 0.2% of the cell population, respectively, resulting in a total apoptotic fraction of 6.8 ± 0.4%. AsA treatment increased early apoptosis to 11.5 ± 0.6% and late apoptosis to 7.2 ± 0.4%, corresponding to 18.7 ± 1.0% total apoptosis.

CDDP treatment produced a more pronounced apoptotic response, with 17.8 ± 1.0% early-apoptotic and 18.6 ± 1.0% late-apoptotic cells, resulting in a total apoptotic fraction of 36.4 ± 2.0%. The highest apoptotic response was observed following combined AsA + CDDP treatment, in which early- and late-apoptotic populations reached 19.5 ± 1.3% and 28.1 ± 1.5%, respectively, yielding 47.6 ± 2.8% total apoptosis (Figure 5). One-way ANOVA followed by Tukey’s multiple-comparison test confirmed that total apoptosis was significantly higher following AsA + CDDP treatment than following CDDP alone (47.6 ± 2.8% vs. 36.4 ± 2.0%; adjusted *p* = 0.000287). The late-apoptotic fraction was also higher with the combination (*p* < 0.001), whereas the early-apoptotic fraction did not differ significantly between these groups (*p* = 0.161).

Correspondingly, the viable cell population decreased from 88.5 ± 0.6% in the control group to 75.0 ± 1.3% with AsA, 54.0 ± 2.6% with CDDP, and 44.5 ± 3.3% with AsA + CDDP. The necrotic cell population was 4.7 ± 0.2%, 6.3 ± 0.3%, 9.6 ± 0.6%, and 7.9 ± 0.5% in the control, AsA, CDDP, and AsA + CDDP groups, respectively. Overall, the combination treatment produced the greatest increase in total apoptosis and was particularly associated with an increase in the late-apoptotic population compared with either single-agent treatment.

### 2.6. Fluorescence Microscopy Confirms Increased Annexin V/PI Staining Following AsA and CDDP Treatment

**Figure 6:**
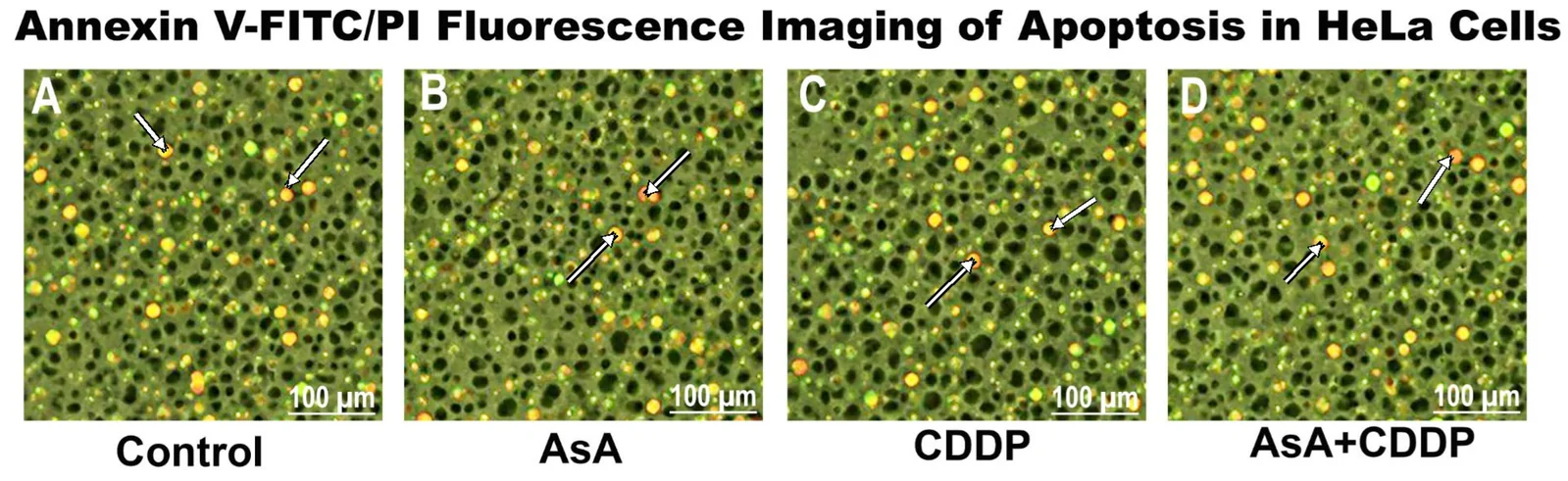
Representative fluorescence microscopy images of Annexin V-FITC/PI-stained HeLa cells following treatment with AsA and CDDP. (**A**) Control cells show predominantly preserved cellular morphology with relatively limited Annexin V-FITC (green) and PI (red/orange) staining. (**B**) AsA treatment is associated with a mild increase in apoptosis-associated fluorescence. (**C**) CDDP treatment produces more prominent PI-positive staining and morphological alterations compared with the control and AsA groups. (**D**) The AsA + CDDP combination shows the most pronounced apoptosis-associated changes, characterized by increased red/orange PI-positive staining and altered cellular morphology. Semiquantitative image analysis showed PI-positive area ratios of 0.17%, 0.22%, 0.36%, and 0.42% in the control, AsA, CDDP, and AsA + CDDP groups, respectively, whereas Annexin V-FITC-positive fluorescence did not differ significantly among the groups. Green: Annexin V-FITC; red/orange: PI. White arrows indicate representative cells showing yellow/orange Annexin V-FITC/PI-associated fluorescence. Scale bar = 100 µm.

The effects of AsA, CDDP, and their combination on apoptosis-associated fluorescence patterns in HeLa cells were further examined by Annexin V-FITC/PI fluorescence microscopy (Figure 6). Control cells exhibited relatively limited Annexin V-FITC (green) and PI (red/orange) fluorescence. AsA treatment produced an increase in apoptosis-associated fluorescence compared with the control, whereas a more pronounced PI-positive signal was observed following CDDP treatment. The AsA + CDDP combination showed the most prominent Annexin V-FITC/PI staining pattern among the experimental groups (Figure 6).

**Figure 7:**
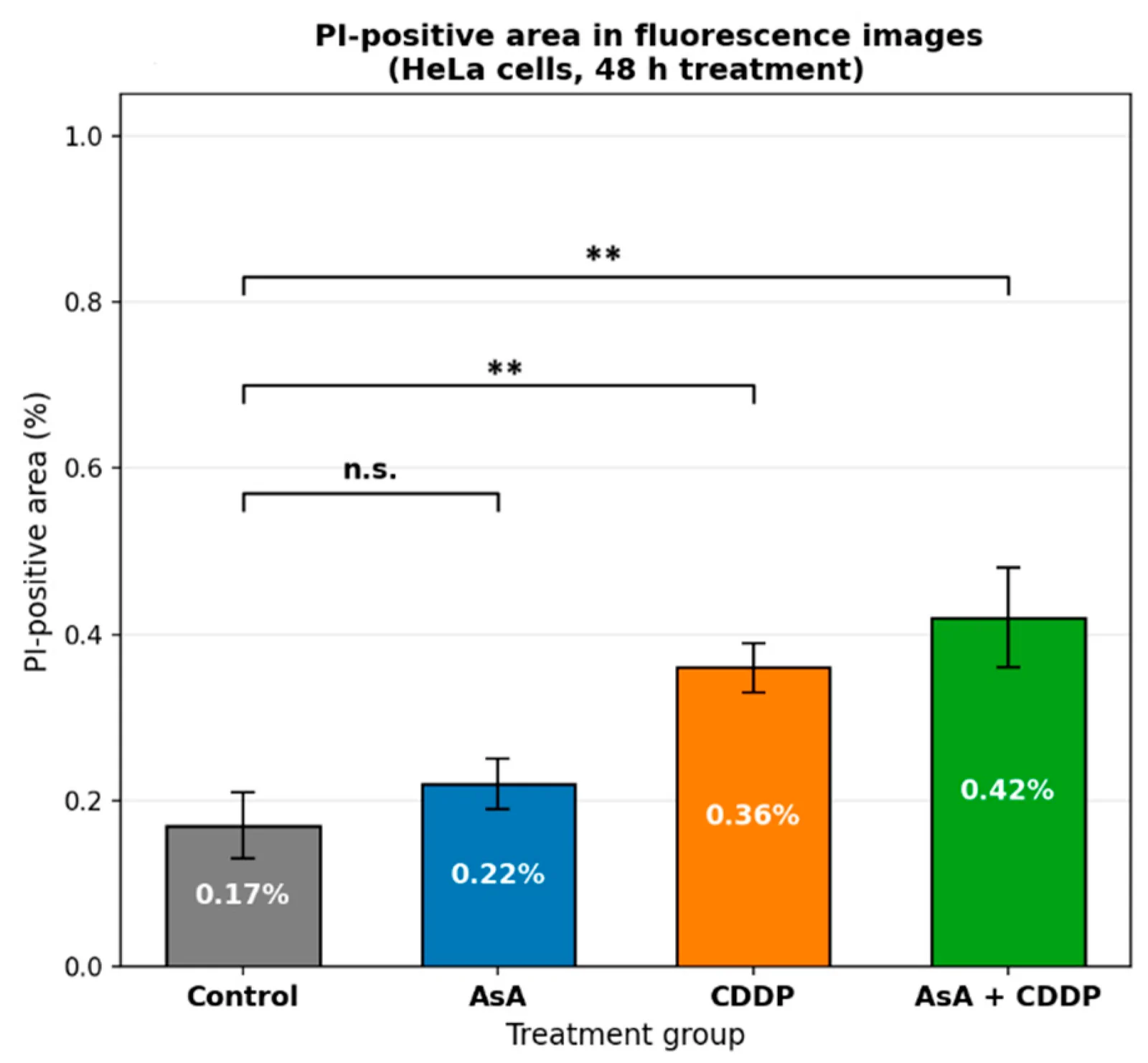
Quantitative analysis of the PI-positive area in fluorescence images of HeLa cells following 48-h treatment with AsA, CDDP, or their combination. The PI-positive area was 0.17% in the control group, 0.22% in the AsA group, 0.36% in the CDDP group, and 0.42% in the AsA + CDDP group. Compared with the control group, the increase was not statistically significant following AsA treatment, whereas significantly higher PI-positive areas were observed following CDDP and AsA + CDDP treatment. Data are presented as mean ± SD (*n* = 3). n.s., not significant; ** *p* < 0.01.

Semiquantitative image analysis showed PI-positive area ratios of 0.17%, 0.22%, 0.36%, and 0.42% in the control, AsA, CDDP, and AsA + CDDP groups, respectively, with the highest PI-positive area observed following combination treatment. In contrast, no statistically significant difference in Annexin V-FITC-positive signal was detected among the groups (*p* > 0.05). These fluorescence microscopy findings were consistent with the increased apoptotic response observed by flow cytometric Annexin V-FITC/PI analysis (Figure 7).

### 2.7. AsA Enhances CDDP-Induced G2/M Cell-Cycle Arrest in HeLa Cells

**Figure 8:**
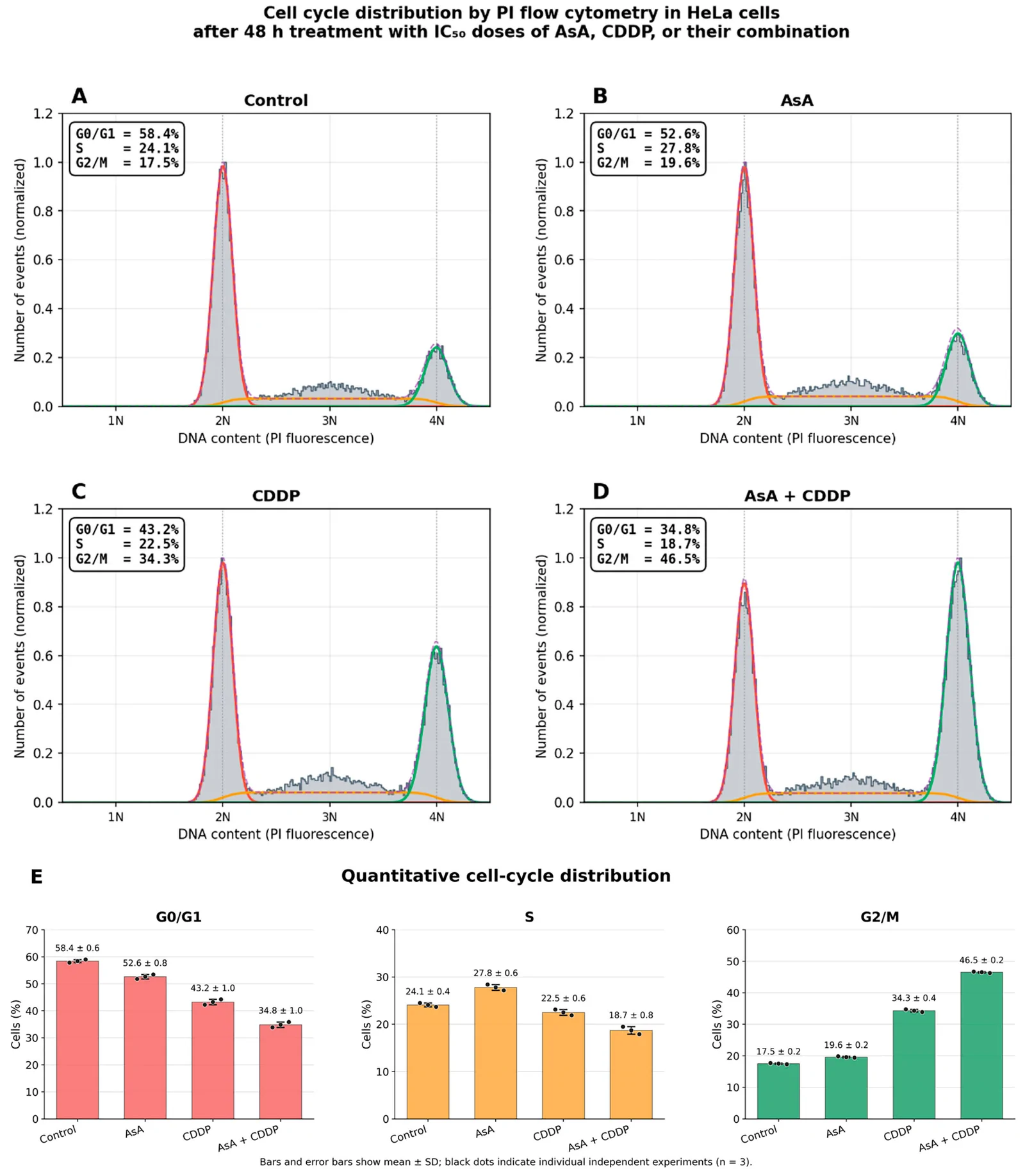
Cell-cycle distribution of HeLa cells following 48-h treatment with AsA, CDDP, or their combination, determined by PI flow cytometry. Representative DNA-content histograms are shown for (**A**) control, (**B**) AsA, (**C**) CDDP, and (**D**) AsA + CDDP groups. The fitted distributions represent the G0/G1 (red), S (orange), and G2/M (green) phases; phase percentages are indicated in each histogram. Vertical dotted lines indicate the 2N and 4N DNA contents. (**E**) Quantitative phase distributions across the four treatment groups. Bars and error bars represent mean ± SD, and black dots indicate individual biological replicates (*n* = 3).

Cell-cycle distribution was evaluated by PI-based flow cytometry following 48-h treatment with AsA, CDDP, or their combination at the corresponding IC_50_-based concentrations (Figure 8). Control HeLa cells showed 58.4 ± 0.6% of cells in G0/G1, 24.1 ± 0.4% in S, and 17.5 ± 0.2% in G2/M phase.

AsA treatment resulted in 52.6 ± 0.8% of cells in G0/G1, 27.8 ± 0.6% in S, and 19.6 ± 0.2% in G2/M. Compared with the control, each phase proportion differed significantly (*p* < 0.001), indicating a modest but statistically significant redistribution of the cell-cycle profile.

CDDP treatment reduced the G0/G1 population to 43.2 ± 1.0% (*p* < 0.001 vs. control) and increased the G2/M population to 34.3 ± 0.4% (*p* < 0.001 vs. control). The S-phase population was 22.5 ± 0.6% and did not differ significantly from the control (*p* = 0.052).

The most pronounced redistribution occurred following combined AsA + CDDP treatment. The G0/G1 population decreased to 34.8 ± 1.0%, the S-phase population to 18.7 ± 0.8%, and the G2/M population increased to 46.5 ± 0.2%. These phase proportions differed from both the control and CDDP-only groups (*p* < 0.001). Thus, the combination produced the greatest accumulation of HeLa cells in G2/M.

### 2.8. Mitochondrial Membrane Potential (ΔΨm)

**Figure 9:**
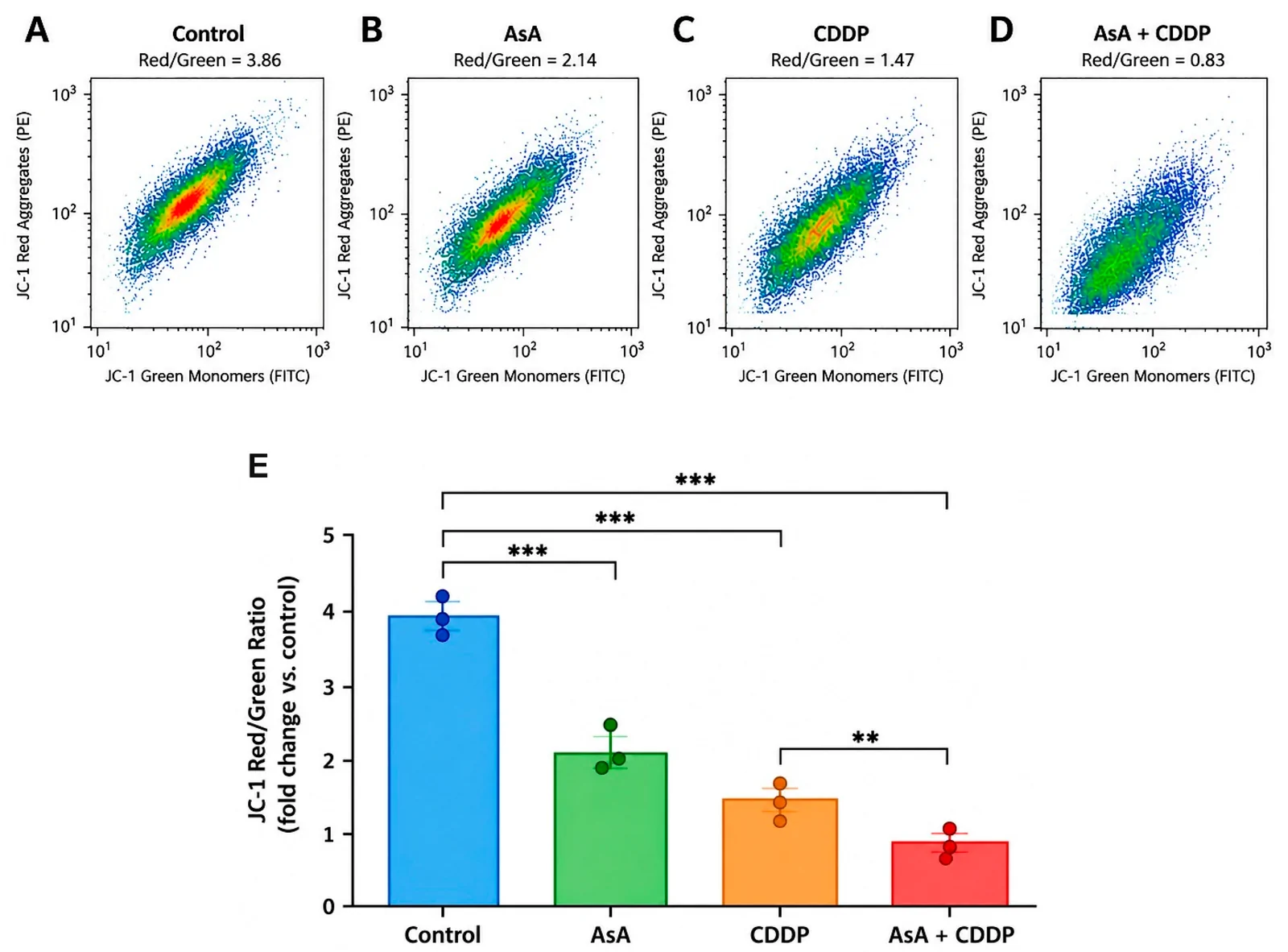
ΔΨm in HeLa cells following AsA and CDDP treatment. (**A**–**D**) Representative JC-1 flow cytometric plots of control, AsA, CDDP, and AsA + CDDP groups, respectively. ΔΨm was assessed based on the JC-1 red/green fluorescence ratio, with a lower ratio indicating mitochondrial membrane depolarization. (**E**) Quantitative analysis of the JC-1 red/green fluorescence ratio. Data are presented as mean ± SD from three independent biological replicates (*n* = 3). ** *p* < 0.01, *** *p* < 0.001. In panels (**A**–**D**), the color gradient from blue to red represents increasing event density in the flow cytometry plots. In panel (**E**), different bar colors distinguish the treatment groups.

JC-1 flow cytometric analysis demonstrated progressive mitochondrial membrane depolarization following treatment with AsA and CDDP, with the greatest reduction in ΔΨm observed in the combination group (Figure 9). Untreated control cells exhibited a high JC-1 red/green fluorescence ratio (3.86 ± 0.24), consistent with predominantly polarized mitochondria.

Treatment with AsA reduced the red/green fluorescence ratio to 2.14 ± 0.18, whereas CDDP treatment resulted in a further decrease to 1.47 ± 0.15. Notably, the AsA + CDDP combination produced the lowest JC-1 red/green fluorescence ratio (0.83 ± 0.09), indicating marked mitochondrial membrane depolarization. The reduction in ΔΨm was significant compared with the untreated control and both single-agent treatment groups (*** *p* < 0.001 vs. control; ** *p* < 0.01 vs. CDDP).

Representative JC-1 flow cytometric plots demonstrated a progressive shift from JC-1 red aggregates toward green monomers following treatment, with the most pronounced shift observed in AsA + CDDP-treated cells. These findings indicate that combined AsA and CDDP treatment induces substantially greater mitochondrial membrane depolarization than either agent alone, supporting the involvement of mitochondrial dysfunction in combination-induced apoptotic cell death.

### 2.9. AsA Enhances CDDP-Induced Caspase-3/7 Activation in HeLa Cells

**Figure 10:**
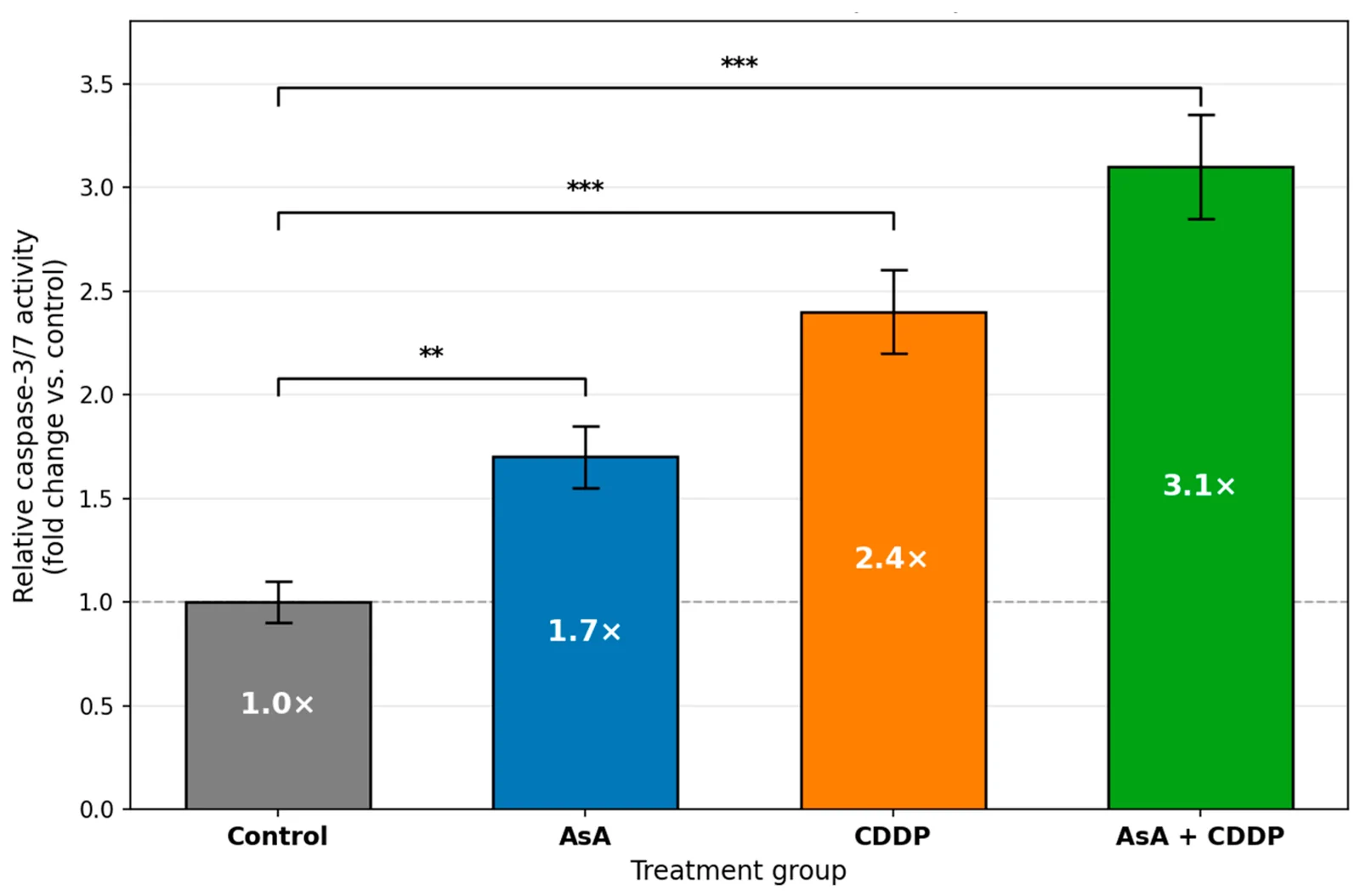
Relative caspase-3/7 activity in HeLa cells following 48-h treatment with AsA, CDDP, or their combination. Caspase-3/7 activity was normalized to the untreated control group (set to 1.0-fold). Relative caspase-3/7 activity increased to approximately 1.7-fold in the AsA group, 2.4-fold in the CDDP group, and 3.1-fold in the AsA + CDDP group. Data are presented as mean ± SD (*n* = 3). ** *p* < 0.01, *** *p* < 0.001 vs. control.

Caspase-3/7 activity was assessed to further evaluate the activation of apoptotic signaling following AsA and CDDP treatment. Relative caspase-3/7 activity was normalized to the untreated control group, which was assigned a value of 1.0-fold (Figure 10). AsA treatment significantly increased caspase-3/7 activity to approximately 1.7-fold compared with the control (** *p* < 0.01), indicating activation of the executioner caspase pathway following AsA exposure.

A greater increase was observed following CDDP treatment, with caspase-3/7 activity reaching approximately 2.4-fold relative to the control (*** *p* < 0.001). The highest activity was detected in the AsA + CDDP group, in which caspase-3/7 activity increased to approximately 3.1-fold compared with the control (*** *p* < 0.001). Thus, the combination produced the greatest activation of caspase-3/7 among the experimental groups.

The progressive increase in caspase-3/7 activity from AsA alone to CDDP and subsequently to the AsA + CDDP combination was consistent with the increased apoptotic response observed by Annexin V-FITC/PI flow cytometry (Figure 5) and the treatment-associated nuclear alterations observed by NucBlue staining (Figure 3 and Figure 4). Collectively, these findings support enhanced activation of executioner caspases in HeLa cells following combined AsA + CDDP treatment.

### 2.10. AsA + CDDP Induces Oxidative Stress, and NAC Partially Rescues Cytotoxicity in HeLa Cells

**Figure 11:**
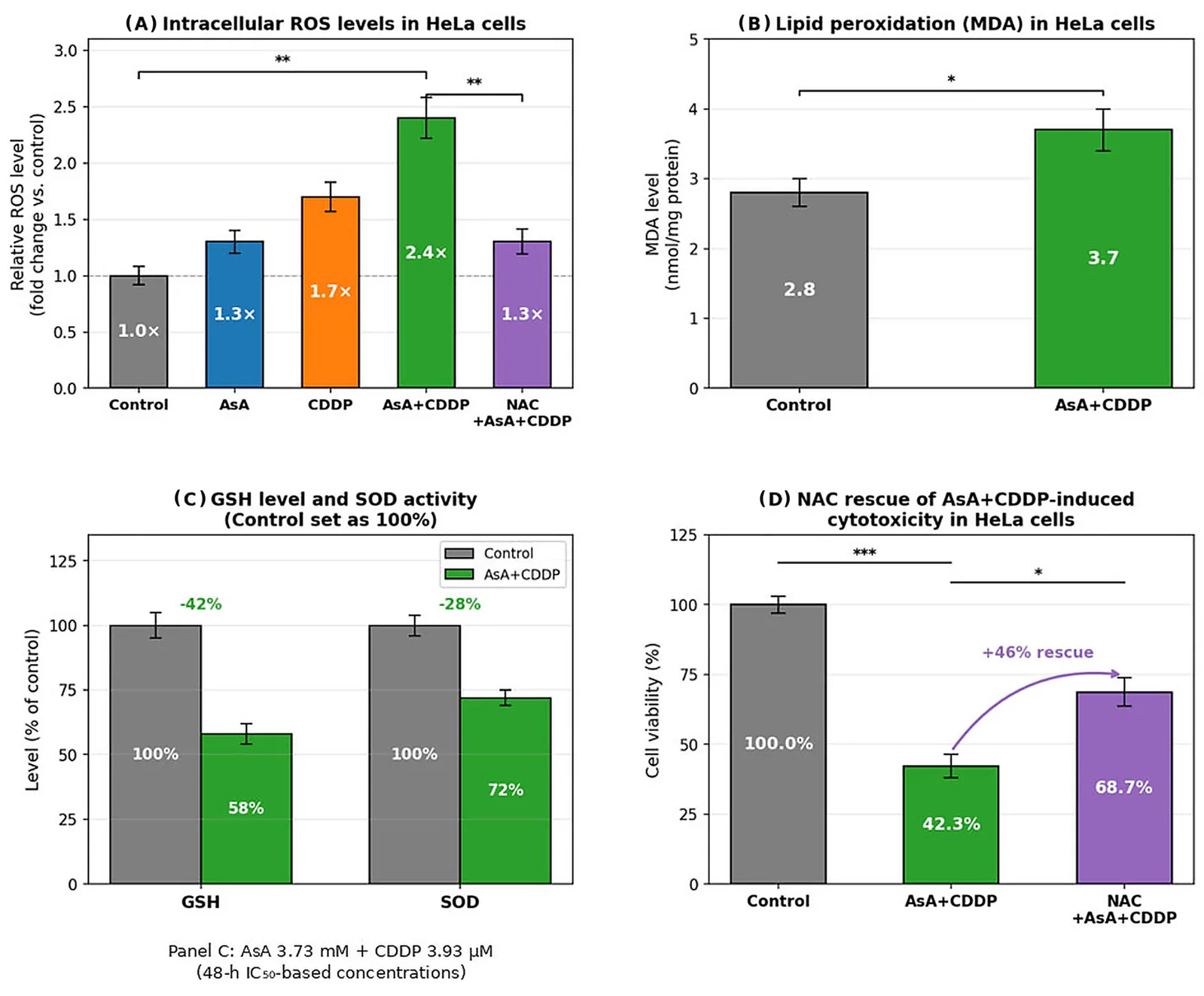
Oxidative stress markers and NAC-mediated rescue in HeLa cells treated with AsA, CDDP, or their combination. HeLa cells were treated for 48 h with AsA, CDDP, or AsA + CDDP at the corresponding 48-h IC_50_-based concentrations, with or without pretreatment with 5 mM NAC. (**A**) Relative intracellular ROS levels measured using DCFH-DA (fold change vs. control). ROS levels increased to 1.3-fold with AsA, 1.7-fold with CDDP, and 2.4-fold with AsA + CDDP, whereas NAC pretreatment reduced the combination-induced ROS level to 1.3-fold. (**B**) MDA levels (nmol/mg protein), an indicator of lipid peroxidation, increased from 2.8 nmol/mg protein in the control group to 3.7 nmol/mg protein in the AsA + CDDP group. (**C**) GSH levels and SOD activity expressed as percentages of the control. AsA + CDDP reduced GSH levels by 42% and SOD activity by 28%. (**D**) Cell viability following NAC pretreatment. With control viability set at 100%, AsA + CDDP reduced cell viability to 42.3%, whereas NAC pretreatment partially restored viability to 68.7%, corresponding to an increase of 26.4 percentage points. Data are presented as mean ± SD (*n* = 3). * *p* < 0.05, ** *p* < 0.01, *** *p* < 0.001.

Intracellular ROS levels increased following AsA and CDDP treatment, with the greatest increase observed in the combination group (Figure 11A). Relative to the control (1.0-fold), ROS levels increased to approximately 1.3-fold with AsA, 1.7-fold with CDDP, and 2.4-fold with AsA + CDDP. The increase induced by the combination was significant compared with the control (** *p* < 0.01). Importantly, NAC pretreatment markedly attenuated the combination-induced increase in ROS, reducing the relative ROS level from 2.4-fold to approximately 1.3-fold (** *p* < 0.01 vs. AsA + CDDP; Figure 11A).

Consistent with increased oxidative stress, lipid peroxidation was enhanced following combination treatment. MDA levels increased from approximately 2.8 nmol/mg protein in control cells to 3.7 nmol/mg protein in the AsA + CDDP group (*p* < 0.05; Figure 11B). In parallel, cellular antioxidant parameters were reduced following combination treatment. GSH levels decreased to 58% of the control level, corresponding to a 42% reduction, whereas SOD activity decreased to 72% of the control level, corresponding to a 28% reduction (Figure 11C).

NAC pretreatment also partially restored cell viability following AsA + CDDP exposure (Figure 11D). Cell viability decreased from 100% in the control group to 42.3% following AsA + CDDP treatment (*p* < 0.001). NAC pretreatment increased viability to 68.7%, which was significantly higher than that observed with AsA + CDDP alone (*p* < 0.05). This represented a 26.4-percentage-point increase, equivalent to recovery of approximately 46% of the viability loss caused by AsA + CDDP. Together, the attenuation of ROS accumulation and partial restoration of cell viability by NAC support the involvement of oxidative stress in AsA + CDDP-induced cytotoxicity.

### 2.11. Effects of AsA + CDDP Treatment on 3D Tumor Spheroid Growth and Viability

**Figure 12:**
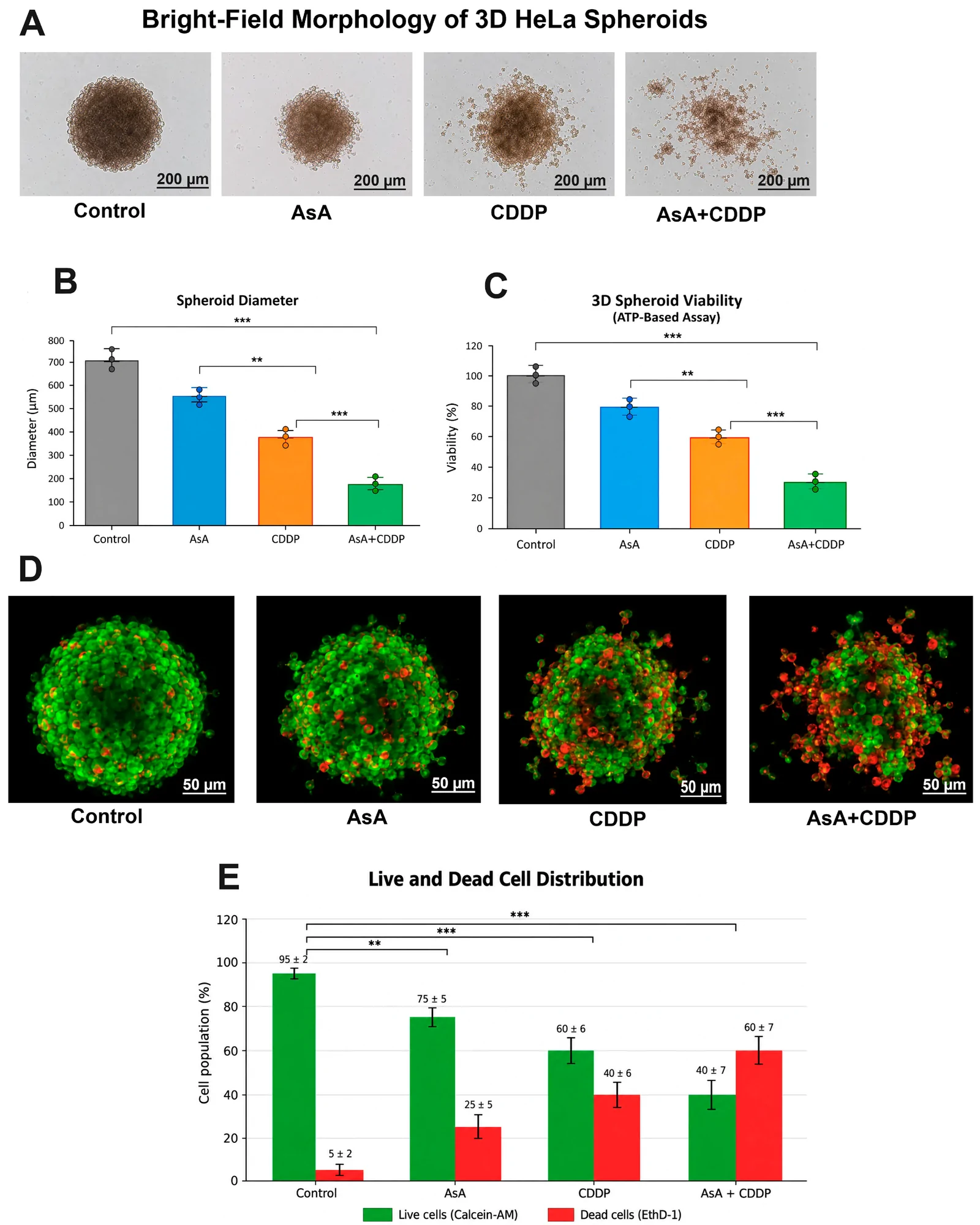
Effects of AsA and CDDP on 3D HeLa spheroid growth and viability. (**A**) Representative bright-field images of control, AsA-, CDDP-, and AsA + CDDP-treated spheroids. Scale bar = 200 μm. (**B**) Quantitative analysis of spheroid diameter. (**C**) Spheroid viability determined using an ATP-based assay. (**D**) Representative Live/Dead fluorescence images showing viable cells stained with Calcein-AM (green) and dead cells stained with EthD-1 (red). Scale bar = 50 μm. (**E**) Quantification of the relative Calcein-AM (green) and EthD-1 (red) fluorescence-signal fractions based on fluorescence intensity; these values do not represent direct counts of viable and dead cells. Data are presented as mean ± SD (*n* = 3). ** *p* < 0.01, *** *p* < 0.001.

The effects of AsA, CDDP, and their combination on HeLa spheroid growth and viability are shown in Figure 12. Bright-field imaging revealed compact and well-defined spheroids in the control group, whereas AsA treatment resulted in a moderate reduction in spheroid size with relatively preserved structural integrity. CDDP caused a more pronounced reduction in spheroid diameter accompanied by irregular borders and peripheral cell dispersion. The AsA + CDDP combination produced the greatest morphological disruption, characterized by marked spheroid disintegration, loss of compactness, and extensive cellular dispersion (Figure 12A).

Quantitative analysis confirmed a progressive reduction in spheroid diameter across the treatment groups. The mean spheroid diameter was approximately 710 µm in the control group, 555 µm following AsA treatment, 380 µm following CDDP treatment, and 175 µm following combined AsA + CDDP treatment. The combination group exhibited the greatest reduction in spheroid diameter (Figure 12B).

Consistent with these morphological findings, ATP-based analysis demonstrated a treatment-dependent decrease in 3D spheroid viability. Viability was approximately 100% in control spheroids and decreased to approximately 80% with AsA and 60% with CDDP treatment. Combined AsA + CDDP treatment further reduced spheroid viability to approximately 30%, representing the strongest cytotoxic effect among the treatment groups (Figure 12C).

Live/Dead fluorescence imaging further demonstrated differences in cellular viability within the spheroids. Control spheroids exhibited predominantly Calcein-AM-positive viable cells with only limited EthD-1-positive dead cells. AsA treatment moderately increased the proportion of dead cells, whereas CDDP produced a more pronounced increase in EthD-1-positive cells together with disruption of spheroid architecture. The AsA + CDDP group showed the greatest loss of viable cells and the highest accumulation of dead cells, accompanied by substantial structural disorganization (Figure 12D).

Quantification of Live/Dead fluorescence images based on signal intensity showed relative Calcein-AM (green) and EthD-1 (red) fluorescence fractions of 95 ± 2% and 5 ± 2%, respectively, in control spheroids. The corresponding green and red signal fractions were 75 ± 5% and 25 ± 5% following AsA treatment, and 60 ± 6% and 40 ± 6% following CDDP treatment. In the AsA + CDDP group, the relative green signal decreased to 40 ± 7%, whereas the red signal increased to 60 ± 7% (Figure 12E). These percentages represent relative fluorescence-signal fractions and should not be interpreted as direct counts of viable and dead cells. Considered alongside spheroid morphology, diameter, and ATP-based viability, the fluorescence patterns were consistent with the strongest treatment effect in the AsA + CDDP group.

### 2.12. AsA + CDDP Modulates Apoptosis- and Proliferation-Related Gene Expression in HeLa Cells

**Figure 13:**
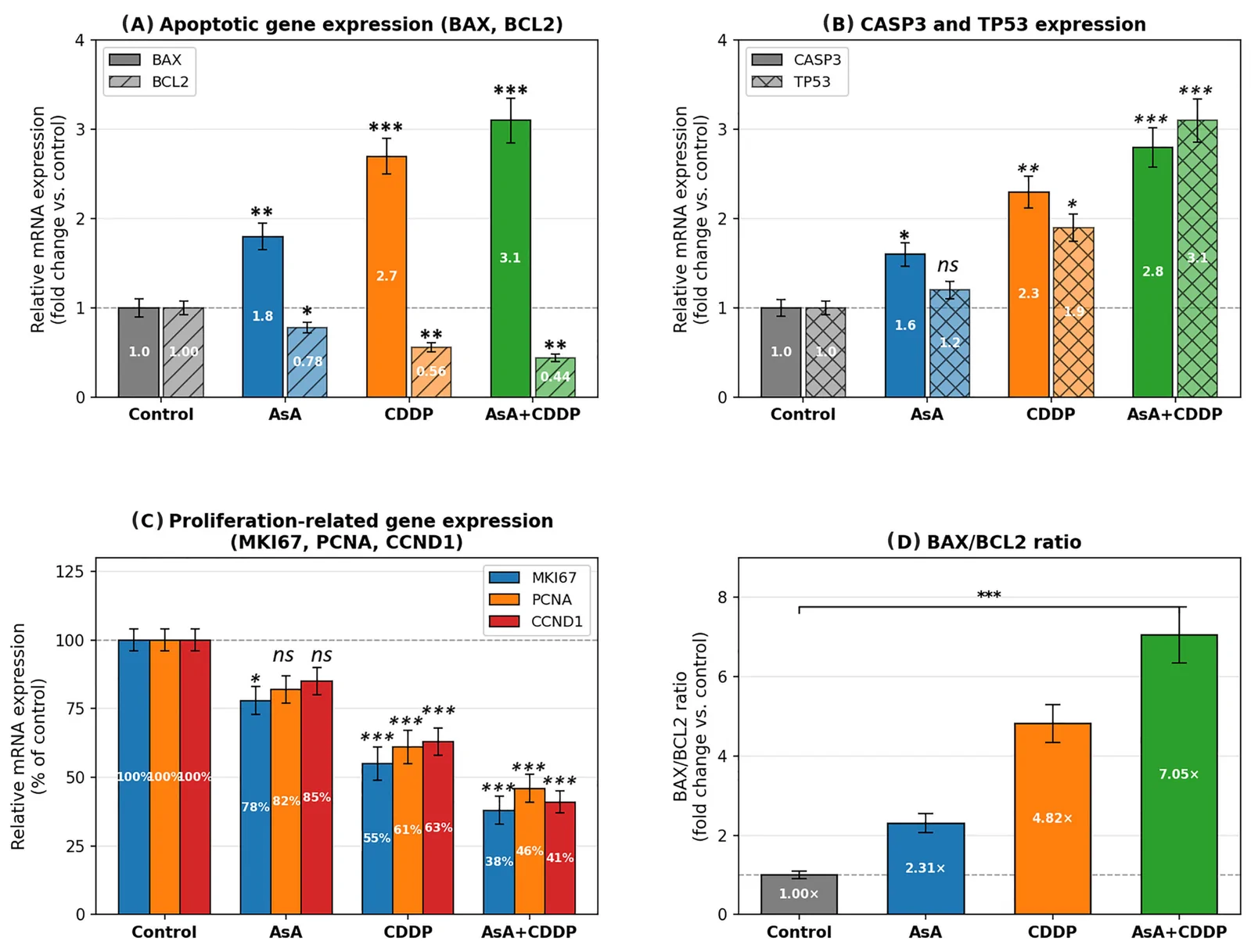
RT-qPCR analysis of apoptosis- and proliferation-related gene expression in HeLa cells following 48-h treatment with AsA, CDDP, or their combination. (**A**) *BAX* and *BCL2* expression. *BAX* expression increased to 1.8-fold with AsA, 2.7-fold with CDDP, and 3.1-fold with AsA + CDDP, whereas *BCL2* expression decreased to 0.78-, 0.56-, and 0.44-fold, respectively. (**B**) *CASP3* and *TP53* expression. *CASP3* expression increased to 1.6-, 2.3-, and 2.8-fold, whereas *TP53* expression reached 1.2-, 1.9-, and 3.1-fold following AsA, CDDP, and AsA + CDDP treatment, respectively. (**C**) Expression of the proliferation-related genes *MKI67*, *PCNA*, and *CCND1*, expressed as a percentage of the control. In the AsA + CDDP group, *MKI67*, *PCNA*, and *CCND1* expression decreased to 38%, 46%, and 41% of control levels, respectively. (**D**) *BAX/BCL2* expression ratio (fold change vs. control), reaching 2.31-fold with AsA, 4.82-fold with CDDP, and 7.05-fold with AsA + CDDP. Data are presented as mean ± SD (*n* = 3). * *p* < 0.05, ** *p* < 0.01, *** *p* **<** 0.001 vs. control.

RT-qPCR analysis revealed treatment-dependent alterations in apoptosis- and proliferation-related gene expression in HeLa cells (Figure 13). *BAX* expression increased from the control level of 1.0-fold to 1.8-fold following AsA treatment (** *p* < 0.01), 2.7-fold following CDDP treatment (*** *p* < 0.001), and 3.1-fold following AsA + CDDP treatment (*** *p* < 0.001). In contrast, *BCL2* expression decreased to 0.78-fold with AsA (* *p* < 0.05), 0.56-fold with CDDP (** *p* < 0.01), and 0.44-fold with AsA + CDDP (** *p* < 0.01) compared with the control (Figure 13A).

Expression of *CASP3* increased to 1.6-fold with AsA (* *p* < 0.05), 2.3-fold with CDDP (** *p* < 0.01), and 2.8-fold with AsA + CDDP (*** *p* < 0.001) (Figure 13B). *TP53* expression was 1.2-fold following AsA treatment, which did not differ significantly from the control, whereas it increased significantly to 1.9-fold with CDDP (* *p* < 0.05) and 3.1-fold with AsA + CDDP (*** *p* < 0.001).

Treatment also reduced the expression of proliferation-related genes (Figure 13C). Following AsA treatment, *MKI67*, *PCNA*, and *CCND1* expression levels were 78%, 82%, and 85% of control, respectively; the reduction was significant only for *MKI67* (* *p* < 0.05). CDDP further reduced their expression to 55%, 61%, and 63%, respectively, with significant reductions in all three genes (*** *p* < 0.001 for *MKI67*; ** *p* < 0.01 for *PCNA*; *** *p* < 0.001 for *CCND1*). The greatest reductions were observed following AsA + CDDP treatment, with *MKI67*, *PCNA*, and *CCND1* expression decreasing to 38%, 46%, and 41% of control, respectively (all *** *p* < 0.001).

Consistent with the reciprocal changes in *BAX* and *BCL2* expression, the *BAX/BCL2* ratio progressively increased from 1.00-fold in control cells to 2.31-fold with AsA, 4.82-fold with CDDP, and 7.05-fold with AsA + CDDP (Figure 13D). The increase in the combination group was significant compared with the control (*** *p* < 0.001). Overall, the combination group exhibited the most pronounced molecular profile, characterized by increased expression of apoptosis-associated genes, suppression of *BCL2*, a marked increase in the *BAX/BCL2* ratio, and reduced expression of proliferation-associated genes.

### 2.13. PPI Network and Functional Enrichment Analyses Identify Apoptosis- and Stress-Related Signaling Pathways

**Figure 14:**
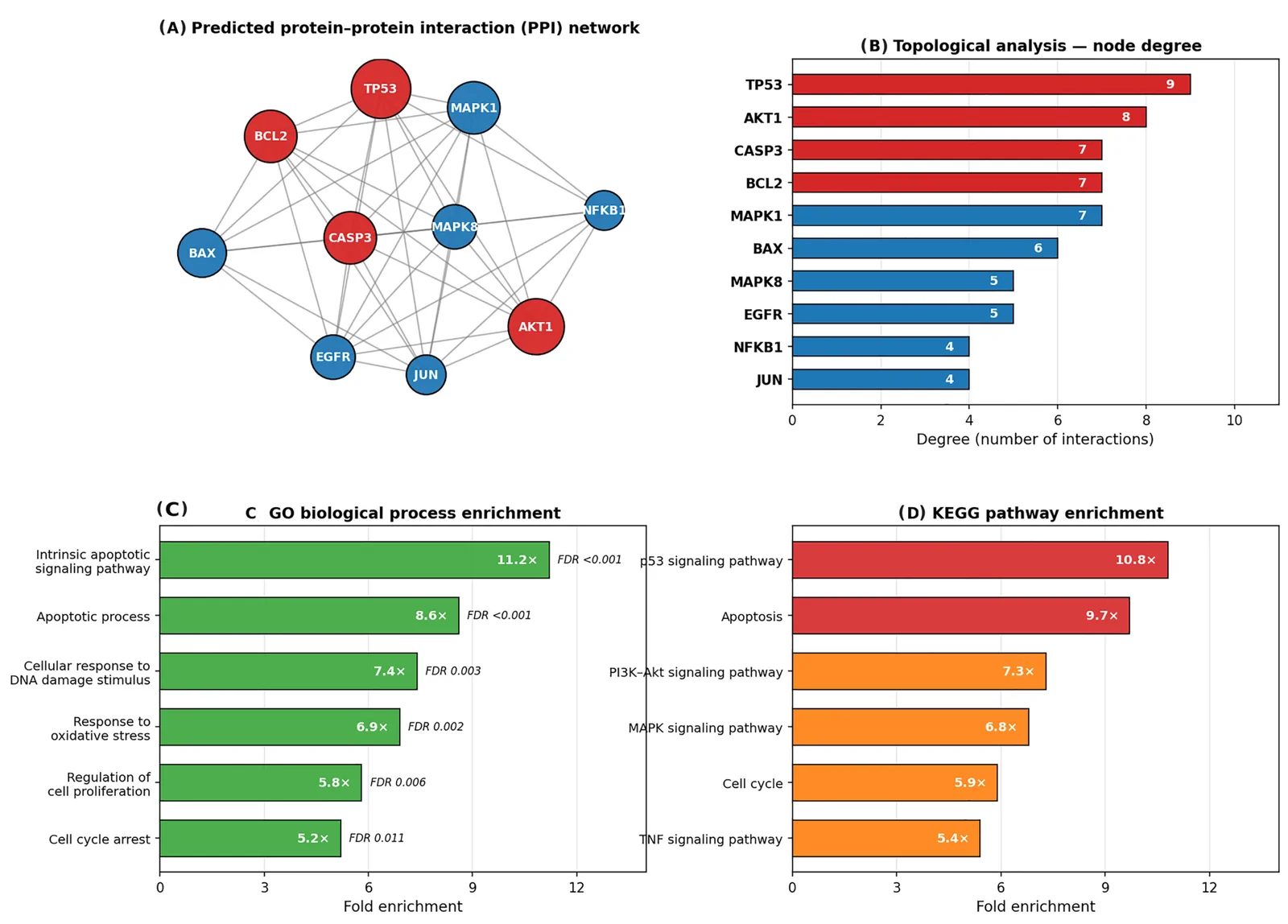
Bioinformatics analysis of selected molecular targets associated with the AsA + CDDP experimental framework. (**A**) Predicted protein–protein interaction (PPI) network constructed using STRING for ten proteins (BAX, BCL2, CASP3, TP53, AKT1, MAPK1, MAPK8, EGFR, NFKB1, and JUN). The network comprised 10 nodes and 42 interactions, compared with approximately 14 interactions expected by chance (PPI enrichment *p* < 0.001). Highly connected proteins, including TP53, AKT1, CASP3, and BCL2, are highlighted in red. (**B**) Topological analysis based on node degree, showing TP53 (9), AKT1 (8), CASP3 (7), BCL2 (7), MAPK1 (7), BAX (6), MAPK8 (5), EGFR (5), NFKB1 (4), and JUN (4). (**C**) Gene Ontology (GO) Biological Process enrichment analysis showing enrichment of the intrinsic apoptotic signaling pathway (11.2-fold, FDR < 0.001), apoptotic process (8.6-fold, FDR < 0.001), cellular response to DNA damage stimulus (7.4-fold, FDR = 0.003), response to oxidative stress (6.9-fold, FDR = 0.002), regulation of cell proliferation (5.8-fold, FDR = 0.006), and cell-cycle arrest (5.2-fold, FDR = 0.011). (**D**) KEGG pathway enrichment analysis showing enrichment of the p53 signaling pathway (10.8-fold), apoptosis (9.7-fold), PI3K–Akt signaling pathway (7.3-fold), MAPK signaling pathway (6.8-fold), cell cycle (5.9-fold), and TNF signaling pathway (5.4-fold).

To explore the molecular context associated with the experimentally investigated targets, a STRING-based protein–protein interaction (PPI) network was constructed using BAX, BCL2, CASP3, TP53, AKT1, MAPK1, MAPK8, EGFR, NFKB1, and JUN. The resulting network comprised 10 nodes and 42 interactions, compared with approximately 14 interactions expected by chance, indicating significant PPI enrichment (*** *p* < 0.001; Figure 14A).

Topological analysis revealed differential connectivity among the network proteins. TP53 showed the highest degree (9), followed by AKT1 (8), CASP3 (7), BCL2 (7), MAPK1 (7), and BAX (6), identifying these proteins as highly connected components of the network. Betweenness centrality analysis further identified TP53 and AKT1 as prominent nodes within the interaction network (Figure 14B).

GO Biological Process enrichment analysis demonstrated significant enrichment of several processes related to the experimental phenotype (Figure 14C). The strongest enrichment was observed for the intrinsic apoptotic signaling pathway (~11.2-fold, FDR < 0.001), followed by the apoptotic process (~8.6-fold, FDR < 0.001), cellular response to DNA damage stimulus (~7.4-fold, FDR = 0.003), response to oxidative stress (~6.9-fold, FDR = 0.002), regulation of cell proliferation (~5.8-fold, FDR = 0.006), and cell-cycle arrest (~5.2-fold, FDR = 0.011). These enriched biological processes corresponded to the major experimental endpoints evaluated in the present study, including oxidative stress, apoptosis, and cell proliferation.

KEGG pathway enrichment analysis further identified the p53 signaling pathway, apoptosis, PI3K–Akt signaling pathway, MAPK signaling pathway, cell cycle, and TNF signaling pathway among the enriched pathways (Figure 14D). Among these, the p53 signaling pathway showed approximately 10.8-fold enrichment, followed by apoptosis (~9.7-fold), PI3K–Akt signaling (~7.3-fold), and MAPK signaling (~6.8-fold). *TP53*, *BAX*, *CASP3*, and *BCL2* were represented among the components associated with the enriched p53/apoptosis-related signaling profile. Overall, the enrichment pattern was consistent with the experimentally observed increases in ROS and apoptosis-associated markers, G2/M accumulation, and suppression of proliferation-related gene expression.

## 3. Discussion

In this study, AsA + CDDP produced synergistic cytotoxicity in HeLa cells under the tested conditions and increased apoptosis, G2/M accumulation, and caspase-3/7 activity. The combination was accompanied by increased ROS and MDA, reduced GSH and SOD activity, decreased ΔΨm, and changes in the expression of *BAX*, *BCL2*, *CASP3*, and *TP53*; NAC partially attenuated ROS accumulation and rescued cell viability. In 3D spheroids, combination treatment caused the greatest structural disruption and reductions in spheroid growth and ATP-based viability, together with an increased relative EthD-1 fluorescence-signal fraction. Overall, the findings support a redox-associated mitochondrial apoptotic response to AsA + CDDP in 2D HeLa cells that was also observed in the 3D spheroid model.

CDDP is a platinum-based chemotherapeutic agent that has long been used in the treatment of cervical cancer. Its principal anticancer activity involves the formation of DNA adducts and intra- and interstrand DNA cross-links, thereby interfering with DNA replication and transcription. Accumulation of unrepaired DNA damage can subsequently induce cell-cycle arrest and apoptotic cell death [25,26]. However, the cytotoxic efficacy of CDDP is influenced by multiple cellular processes, including DNA damage responses, DNA repair capacity, anti-apoptotic signaling, and cellular redox homeostasis [27]. The pronounced reduction in HeLa cell viability observed following CDDP treatment in the present study is consistent with these established mechanisms. Furthermore, the substantial G2/M accumulation observed following CDDP exposure supports disruption of cell-cycle progression as an important component of the cellular response.

The biological activity of AsA in cancer cells is strongly concentration-dependent. Although AsA predominantly acts as an antioxidant at physiological concentrations, pharmacological concentrations can exert pro-oxidant effects and promote hydrogen peroxide generation [28,29]. Chen et al. demonstrated that pharmacological AsA concentrations could inhibit tumor growth through oxidative mechanisms [30], while Testa et al. highlighted the potential of high-dose AsA to exploit redox vulnerabilities of malignant cells [31]. In the present study, AsA reduced HeLa cell viability and increased intracellular ROS, particularly at higher concentrations, consistent with a concentration-dependent pro-oxidant effect. The accompanying increases in apoptotic parameters and changes in mitochondrial function further suggest that the biological effects of pharmacological AsA extend beyond a simple antioxidant response.

One of the principal findings of the present study was the enhanced cytotoxic response produced by combined AsA and CDDP treatment. After 48 h, the combination reduced HeLa cell viability to approximately 42%, representing a more pronounced effect than either agent alone. CI analysis yielded a CI value of 0.72 in HeLa cells, consistent with a synergistic interaction under the tested experimental conditions. In contrast, the CI value of 0.98 observed in HaCaT cells was within the nearly additive range. These findings suggest that the nature and magnitude of the interaction may differ according to cellular context. Nevertheless, synergy is dependent on experimental variables including dose ratio, exposure duration, and the analytical model used; therefore, the observed interaction should be interpreted specifically within the treatment conditions examined in the present study.

The literature regarding interactions between AsA and chemotherapeutic agents is not entirely consistent. Although pharmacological AsA has been reported to enhance anticancer treatment responses in several experimental settings [32], previous studies in cervical cancer models have also examined its interaction with cisplatin. Reddy et al. reported that vitamin C stabilized p53 and sensitized HeLa cells to cisplatin-induced cell-cycle arrest and apoptosis [15]. Wu et al. found that pharmacological ascorbate increased the susceptibility of HeLa cells to cisplatin [16], while Leekha et al. observed enhanced cell death, hydrogen peroxide generation, and p53 upregulation following vitamin C plus cisplatin treatment in SiHa cells [17]. Thus, the interaction between AsA and CDDP should not be interpreted simply as antioxidant supplementation. AsA concentration, treatment sequence, tumor-cell phenotype, metabolic state, and intracellular redox conditions may influence the resulting response. In the present study, AsA + CDDP produced a synergistic interaction in HeLa cells (CI = 0.72), whereas the interaction was nearly additive in HaCaT cells (CI = 0.98), supporting the importance of these context-dependent effects.

Oxidative stress appears to contribute to the enhanced response to AsA + CDDP. Intracellular ROS increased approximately 2.4-fold following combination treatment, exceeding the increases observed with either agent alone. CDDP has previously been associated with mitochondrial dysfunction and increased ROS generation, which can contribute to its cytotoxic activity [33,34]. NAC pretreatment reduced combination-induced ROS from approximately 2.4-fold to 1.3-fold and partially restored cell viability from 42.3% to 68.7%, supporting a contribution of oxidative stress to cytotoxicity. However, because NAC can also affect glutathione metabolism and cellular redox homeostasis, this partial rescue does not establish ROS as the sole mediator [35]. The redox-marker results were consistent with this response: MDA increased from approximately 2.8 to 3.7 nmol/mg protein, while GSH and SOD activity decreased to 58% and 72% of control, respectively. Oxidative stress can damage DNA, proteins, and membrane lipids and contribute to cell-death pathways [36]. JC-1 analysis showed the greatest decrease in the red/green fluorescence ratio following combination treatment, indicating mitochondrial depolarization and loss of ΔΨm; this ratio reflects the potential-dependent formation of red-emitting JC-1 aggregates in polarized mitochondria [37]. Together, the ROS increase, NAC-mediated partial rescue, redox-marker changes, and mitochondrial depolarization support an association between oxidative stress and mitochondrial dysfunction during AsA + CDDP-induced cell death.

The apoptosis findings were consistent with this interpretation. Annexin V-FITC/PI analysis demonstrated a progressive increase in apoptotic cells from 6.8% in the control group to 18.7% with AsA, 36.4% with CDDP, and 47.6% following combination treatment. The fluorescence microscopy and NucBlue nuclear morphology findings further supported treatment-associated apoptotic alterations. Regulation of the BAX/BCL2 balance constitutes an important control point in the intrinsic mitochondrial apoptotic pathway [38,39]. In the present study, *BAX* expression increased while *BCL2* expression decreased, resulting in an approximately 7.05-fold increase in the *BAX*/*BCL2* ratio following combination treatment. When considered alongside the pronounced reduction in ΔΨm demonstrated by JC-1 staining, these reciprocal changes provide complementary evidence supporting activation of mitochondrial apoptotic signaling.

The increase in caspase-3/7 activity further supports this mechanism. Caspase-3/7 represents a major executioner component of apoptotic cell death and contributes to the characteristic biochemical and morphological alterations associated with apoptosis [40]. In the present study, the combination increased caspase-3/7 activity to approximately 3.1-fold relative to the control, while *CASP3* expression increased approximately 2.8-fold. The parallel increase in *CASP3* transcription and caspase-3/7 activity suggests enhancement of apoptotic signaling at both transcriptional and functional levels. Taken together with the increased *BAX/BCL2* ratio, mitochondrial depolarization, Annexin V-FITC/PI findings, and apoptotic nuclear alterations, these observations support coordinated activation of the intrinsic apoptotic machinery following AsA + CDDP exposure.

Cell-cycle and proliferation-related findings provide an additional dimension to the combination response. CDDP increased the proportion of cells in G2/M, and this effect became substantially more pronounced following combined treatment, with approximately 46.5% of cells accumulating in G2/M compared with 17.5% in the control group. This alteration was accompanied by suppression of the proliferation-associated genes *MKI67*, *PCNA*, and *CCND1*. PCNA is closely associated with DNA replication and proliferative activity, whereas cyclin D1 plays an important role in regulation of cell-cycle progression [41]. The simultaneous G2/M accumulation and downregulation of these proliferation-associated markers indicate that AsA + CDDP not only promotes apoptotic cell death but also markedly disrupts proliferative and cell-cycle processes. The increase in *TP53* expression, which reached approximately 3.1-fold following combination treatment, is also consistent with activation of cellular stress and cell-cycle regulatory responses.

Chemosensitization to platinum-based drugs has been associated with distinct molecular mechanisms in different cancer models. In wild-type EGFR non-small-cell lung cancer cells, cudratricusxanthone A enhanced cisplatin sensitivity by binding to EGFR and suppressing EGFR/ERK/AKT signaling [42]. In colorectal cancer cells, salinomycin enhanced oxaliplatin activity by increasing ROS, decreasing ΔΨm, and activating the MAPK pathway; NAC pretreatment reversed these effects [43]. A different mechanism was reported for Cathayanon E, which enhanced oxaliplatin sensitivity in colorectal cancer by binding to MCL1 and promoting its β-TRCP-mediated ubiquitination and proteasomal degradation, thereby inducing mitochondrial apoptotic signaling [44]. Together, these studies illustrate distinct routes to platinum-drug sensitization, involving suppression of EGFR-dependent survival signaling, ROS-associated mitochondrial and MAPK responses, or degradation of an anti-apoptotic protein. In our HeLa model, increased ROS, partial NAC-mediated rescue, and decreased ΔΨm are consistent with a redox-associated mitochondrial response to AsA + CDDP. Because EGFR/ERK/AKT, MAPK, and MCL1 were not directly assessed in the present study, their involvement in this response remains undetermined.

An important extension of the present findings was the evaluation of the AsA + CDDP response in a 3D HeLa spheroid model. Three-dimensional tumor models provide a more spatially organized cellular environment than conventional monolayer cultures and introduce additional constraints on treatment response through cell–cell interactions and gradients within the spheroid. In the present study, the enhanced activity of AsA + CDDP observed in 2D cultures was retained under 3D growth conditions. Combination treatment produced the most pronounced disruption of spheroid morphology and integrity, accompanied by reductions in spheroid growth and ATP-based viability and an increased relative EthD-1 red fluorescence-signal fraction in the Live/Dead analysis. Because this fluorescence-based fraction does not directly quantify cell number, it was interpreted as supportive signal-based evidence alongside spheroid morphology and ATP-based viability. Multicellular spheroids have been used to combine morphological assessment, vital-dye imaging, and ATP-based viability measurements when evaluating drug responses in 3D cultures [45]. The use of these complementary endpoints in the present study therefore allowed the combination response to be assessed in terms of both spheroid structure and viability. These findings are particularly relevant because they demonstrate that the enhanced treatment response was not restricted to conventional monolayer conditions. Previous studies from our group using combined natural compounds and CDDP or other chemotherapeutic agents have similarly emphasized the value of evaluating treatment responses across both 2D and 3D tumor models [5,33]. Nevertheless, spheroids remain simplified in vitro models and cannot reproduce all components of the in vivo tumor microenvironment.

The differences observed between HeLa and HaCaT cells are also relevant. HeLa cells exhibited lower estimated IC_50_ values for both agents at 48 h than HaCaT cells, and CI analysis indicated synergy in HeLa cells but a nearly additive interaction in HaCaT cells. HeLa cells are HPV-18-positive, and viral E6/E7 oncoproteins disrupt important tumor-suppressor mechanisms involving p53 and retinoblastoma proteins [46]. Such molecular characteristics may contribute to differences in cellular responses to oxidative stress, DNA damage, and chemotherapeutic exposure. However, the present findings establish differences in sensitivity between the two experimental cell models rather than definitive tumor selectivity.

This distinction is important because HaCaT cells represent a spontaneously immortalized human keratinocyte model and should not be regarded as equivalent to normal cervical epithelium [22]. Consequently, the greater sensitivity observed in HeLa cells should not be interpreted as conclusive evidence of selective toxicity toward cervical cancer cells. Validation using primary cervical epithelial cells or other appropriate non-malignant cervical cellular models would allow a more rigorous assessment of the therapeutic selectivity and safety window of the AsA + CDDP combination.

Bioinformatics analyses provided a complementary framework for interpreting the experimental findings. The PPI network identified TP53, AKT1, CASP3, BCL2, MAPK1, and BAX among the highly connected proteins, linking cellular stress responses, survival signaling, and apoptotic regulation. Network-based approaches such as STRING facilitate interpretation of experimentally investigated targets within broader biological interaction networks [47,48]. GO enrichment highlighted intrinsic apoptotic signaling, apoptosis, cellular responses to DNA damage and oxidative stress, regulation of proliferation, and cell-cycle arrest, whereas KEGG analysis identified p53, apoptosis, PI3K–Akt, MAPK, cell-cycle, and TNF signaling pathways. These enriched processes were broadly consistent with the experimentally observed ROS accumulation, mitochondrial dysfunction, G2/M accumulation, altered *BAX/BCL2* balance, increased *CASP3* expression and caspase-3/7 activity, and suppression of proliferation-associated genes. However, because the bioinformatics analysis was performed using a predefined set of selected proteins, these findings should be regarded as supportive and hypothesis-generating rather than as independent identification or experimental validation of molecular targets.

The present findings are also consistent with our previous studies investigating natural compound–chemotherapeutic combinations across different cancer models. In OVCAR3 ovarian cancer cells, combined thymoquinone and docetaxel treatment enhanced cytotoxic and apoptotic responses, increased ROS accumulation, and produced pronounced growth inhibition and structural disruption in 3D tumor spheroids [49]. Similarly, in 4T1 breast cancer cells, rosmarinic acid combined with doxorubicin enhanced apoptosis, ROS accumulation, mitochondrial depolarization, and G2/M-phase accumulation, while NAC partially reversed ROS elevation and treatment-associated cytotoxicity in both monolayer and 3D spheroid models [50]. In MDA-MB-231 triple-negative breast cancer cells, thymoquinone potentiated docetaxel-induced cytotoxicity and apoptosis, accompanied by increased ROS generation, partial NAC-mediated rescue, enhanced intrinsic apoptotic signaling, and modulation of PI3K/AKT pathway-related gene expression [51]. Although these studies involved different tumor types, natural compounds, and chemotherapeutic agents, their findings converge with the present results in demonstrating enhanced combination-induced cytotoxicity associated with oxidative stress and apoptotic signaling. Importantly, the current study extends this experimental framework to the AsA + CDDP combination in cervical cancer cells and further integrates mitochondrial membrane depolarization and 3D spheroid responses into the observed mechanistic profile.

Several limitations should nevertheless be considered. First, although the inclusion of both 2D monolayer cultures and 3D HeLa spheroids strengthens the experimental design, in vitro findings cannot be directly extrapolated to clinical efficacy or therapeutic benefit. The 3D spheroid system also does not reproduce the complete cervical tumor microenvironment, particularly stromal, vascular, and immune components. Furthermore, the AsA concentrations used were within the pharmacological range and therefore should not be considered equivalent to exposure achieved through standard oral vitamin C administration [7]. Second, HaCaT cells are spontaneously immortalized keratinocytes and are not equivalent to normal cervical epithelial cells [22]; consequently, additional non-malignant cervical models are required before conclusions regarding tumor selectivity can be established. Third, although JC-1 analysis demonstrated mitochondrial depolarization and NAC rescue supported a functional contribution of oxidative stress, additional assessment of mitochondrial apoptotic events, including cytochrome ***c*** release and upstream caspase activation, would further clarify the sequence of molecular events. Fourth, the present study evaluated cisplatin, a Pt(II) agent, and did not include a Pt(IV) compound such as satraplatin for comparison. Although ascorbate can reduce selected Pt(IV) complexes, the reduction kinetics depend on the specific complex and chemical environment; plasma thiols may also react more rapidly than ascorbate under some conditions [19]. Because platinum speciation and reduction kinetics were not measured, our findings cannot establish direct AsA–platinum chemical interactions, Pt(IV) activation by AsA, or comparative efficacy or safety relative to Pt(IV)-based agents. Future studies should directly compare Pt(II) and Pt(IV) compounds and assess platinum speciation and reduction kinetics. Finally, the bioinformatics analyses were based on selected proteins and database-derived associations and therefore do not constitute direct experimental validation of the predicted protein–protein interactions or signaling pathways.

## 4. Materials and Methods

### 4.1. Cell Culture and Maintenance

The human cervical cancer cell line HeLa and the human keratinocyte cell line HaCaT were used in this study. HeLa cells were obtained from the American Type Culture Collection (ATCC, Manassas, VA, USA), whereas HaCaT cells were obtained from Cell Lines Service (CLS; catalog no. 300493, Eppelheim, Germany). Cells were maintained in Dulbecco’s Modified Eagle Medium (DMEM; Gibco, Thermo Fisher Scientific, Waltham, MA, USA) supplemented with 10% fetal bovine serum (FBS; Gibco, Thermo Fisher Scientific, Waltham, MA, USA) and 1% penicillin–streptomycin (Gibco, Thermo Fisher Scientific, Waltham, MA, USA).

Cells were cultured at 37 °C in a humidified atmosphere containing 5% CO_2_. The culture medium was replaced every 2–3 days, and cells were subcultured at approximately 80% confluence using 0.25% trypsin–EDTA (Gibco, Thermo Fisher Scientific, Waltham, MA, USA). Cells between passages 3 and 10 were used for all experiments. Cell viability was assessed before experimental procedures, and only cultures exhibiting >90% viability were included. Cells were routinely examined for morphological integrity and signs of contamination throughout the experimental period.

### 4.2. Chemicals and Treatment Compounds

L-Ascorbic acid (AsA; ≥99% purity) and cisplatin (CDDP; Calbiochem^®^, catalog no. 232120; ≥98% purity by HPLC) were obtained from Sigma-Aldrich (Merck KGaA, Darmstadt, Germany). AsA solutions were freshly prepared in sterile distilled water immediately before each experiment and diluted with culture medium to the required working concentrations. CDDP was dissolved in sterile distilled water containing 154 mM NaCl (0.9% *w*/*v*) to prepare a stock solution and was subsequently diluted in culture medium to the required final concentrations immediately before treatment. All working solutions were prepared under sterile conditions.

### 4.3. Experimental Design and Drug Treatment

HeLa and HaCaT cells were assigned to four experimental groups: untreated control, AsA, CDDP, and combined AsA + CDDP treatment. For dose–response experiments, cells were exposed to AsA at concentrations of 0.1, 0.5, 1, 2.5, 5, and 10 mM or CDDP at concentrations of 0.5, 1, 2.5, 5, 10, and 20 µM for 24 or 48 h.

For combination experiments, AsA and CDDP concentrations were selected on the basis of the dose–response curves obtained from the single-agent experiments. The agents were administered simultaneously using paired-dose combinations selected on the basis of the single-agent dose–response curves, and their interaction was subsequently evaluated using the Chou–Talalay CI method. For downstream mechanistic experiments, cells were assigned to the control, AsA, CDDP, and AsA + CDDP groups and treated using the corresponding 48-h IC_50_-based concentrations determined from the viability experiments. Unless otherwise specified for individual assays, treatments were maintained for 48 h.

Control cells were cultured under identical experimental conditions without exposure to AsA or CDDP. Because both compounds were prepared in aqueous vehicles, no organic solvent vehicle was introduced into the experimental groups. All experiments were performed using three independent biological replicates, with technical replicates included as specified for the individual assays.

### 4.4. Assessment of Cell Viability by XTT Assay

Cell viability was assessed using the XTT [2,3-bis(2-methoxy-4-nitro-5-sulfophenyl)-2H-tetrazolium-5-carboxanilide] assay [52]. HeLa and HaCaT cells were seeded into 96-well culture plates at a density of 5 × 10^3^ cells/well in 100 µL of complete culture medium and allowed to adhere for 24 h at 37 °C in a humidified atmosphere containing 5% CO_2_. Cells were subsequently treated with increasing concentrations of AsA (0.1, 0.5, 1, 2.5, 5, and 10 mM) or CDDP (0.5, 1, 2.5, 5, 10, and 20 µM), as well as their combinations, and incubated for 24 or 48 h.

Because AsA may contribute to background absorbance in tetrazolium-based assays, cell-free wells containing the corresponding AsA concentrations were included for background correction. Untreated cells maintained in complete culture medium served as the control. Following treatment, 50 µL of XTT/electron-coupling reagent mixture containing phenazine methosulfate (PMS), prepared according to the manufacturer’s instructions, was added to each well. Plates were incubated for an additional 2 h at 37 °C in the dark, and the absorbance of the resulting formazan product was measured at 570 nm using a microplate reader.

For AsA-containing experimental conditions, the absorbance obtained from the corresponding cell-free AsA blank was subtracted to minimize compound-associated background interference. Cell viability was calculated relative to untreated control cells according to the following equation:

Cell viability (%) = [(A_exp − A_blank)/(A_control − A_blank)] × 100

Here, A_exp represents the absorbance of treated cells, A_blank represents the corresponding cell-free background absorbance, and A_control represents the absorbance of untreated control cells after blank correction. For each independent biological experiment, absorbance values from the duplicate technical wells were averaged at each concentration, and the resulting concentration–response data for AsA and CDDP were fitted separately using nonlinear regression with a four-parameter variable-slope model in GraphPad Prism (version 10.0). IC_50_ values were estimated for each independent experiment and are reported as the mean ± SD of three independent experiments (*n* = 3).

Experiments were performed in three independent biological replicates, with duplicate technical wells for each experimental condition within each independent experiment.

### 4.5. Drug Interaction Analysis Using the Chou–Talalay CI

The interaction between AsA and CDDP was quantitatively evaluated using the Chou–Talalay CI method. Dose–response data obtained from the XTT viability assay for AsA and CDDP administered individually and in combination were analyzed using CompuSyn software  (version 1.0; ComboSyn Inc., Paramus, NJ, USA).. The fraction affected (Fa), representing the proportion of growth inhibition relative to untreated control cells, was calculated from the viability data and used for median-effect and CI analyses. CI values were determined at the corresponding effect levels for the AsA + CDDP combinations. CI values were interpreted according to the Chou–Talalay classification as follows: CI < 0.10, very strong synergism; 0.10–0.30, strong synergism; 0.30–0.70, synergism; 0.70–0.85, moderate synergism; 0.85–0.90, slight synergism; 0.90–1.10, nearly additive; 1.10–1.20, slight antagonism; 1.20–1.45, moderate antagonism; 1.45–3.30, antagonism; 3.30–10.00, strong antagonism; and >10.00, very strong antagonism. CI analyses were performed separately for HeLa and HaCaT cells to compare the interaction profile of the combination between cancer and non-cancerous cellular models.

### 4.6. NucBlue Staining and Semiquantitative Analysis of Nuclear Morphology

Apoptosis-associated nuclear morphological alterations were evaluated in HeLa cells using NucBlue (Hoechst 33342) nuclear staining. Following 48-h treatment with AsA, CDDP, or the AsA + CDDP combination at the corresponding 48-h IC_50_-based concentrations, cells were stained with NucBlue according to the manufacturer’s instructions and examined by fluorescence microscopy. Untreated cells served as the control. Representative images were acquired at ×10 magnification under identical imaging conditions across all experimental groups.

Nuclear morphology was evaluated based on chromatin condensation, increased nuclear fluorescence intensity, nuclear irregularity, and fragmentation. For semiquantitative analysis, nuclei were scored on a five-point scale from 0 to 4 according to the extent of apoptosis-associated morphological alterations: score 0, morphologically normal nuclei with 0–5% condensation; score 1, mild chromatin condensation (6–25%); score 2, moderate condensation with chromatin marginalization (26–50%); score 3, severe condensation with initial nuclear fragmentation (51–75%); and score 4, extensive nuclear fragmentation/karyorrhexis (>75%). The mean nuclear apoptosis score and the percentage distribution of nuclei across the five score categories were calculated for each experimental group. Analyses were performed in three independent biological experiments.

### 4.7. Flow Cytometric Analysis of Apoptosis

Apoptotic cell death was assessed using Annexin V-FITC/propidium iodide (PI) staining. Following treatment, both adherent and detached cells were collected, washed twice with cold phosphate-buffered saline (PBS), and resuspended in 1× Annexin V binding buffer. Cells were stained using an Annexin V-FITC Apoptosis Detection Kit (BD Biosciences, San Jose, CA, USA) according to the manufacturer’s instructions. Samples were incubated with Annexin V-FITC and PI in the dark at room temperature and subsequently analyzed using a BD FACSCanto II flow cytometer (BD Biosciences, San Jose, CA, USA).

For each sample, at least 20,000 events were acquired. Cells were classified according to Annexin V-FITC and PI staining as viable (Annexin V^−^/PI^−^), early apoptotic (Annexin V^+^/PI^−^), late apoptotic (Annexin V^+^/PI^+^), or necrotic (Annexin V^−^/PI^+^). Total apoptotic cells were calculated as the sum of the early- and late-apoptotic populations. Data were obtained from three independent biological experiments.

### 4.8. Annexin V-FITC/PI Fluorescence Microscopy and Image Quantification

Apoptosis-associated fluorescence patterns were further evaluated in HeLa cells using Annexin V-FITC/PI staining followed by fluorescence microscopy. Following 48-h treatment with AsA, CDDP, or the AsA + CDDP combination at the corresponding 48-h IC_50_-based concentrations, cells were washed with phosphate-buffered saline (PBS) and stained with Annexin V-FITC and PI according to the manufacturer’s instructions. Untreated cells served as the control. Fluorescence images were acquired under identical imaging and exposure conditions across all experimental groups to ensure comparability.

Annexin V-FITC fluorescence was visualized in the green channel, whereas PI-positive cells were detected in the red/orange channel. Representative images were evaluated for treatment-associated alterations in fluorescence patterns and cellular morphology. For semiquantitative image analysis, Annexin V-FITC-positive and PI-positive fluorescence signals were quantified from images acquired under identical settings. The PI-positive area was calculated relative to the total analyzed image area and expressed as a percentage. Annexin V-FITC-positive fluorescence was similarly quantified for comparison among experimental groups. Image-based measurements were obtained from three independent biological experiments, and the resulting values were used for statistical analysis.

### 4.9. Flow Cytometric Analysis of Cell-Cycle Distribution

Cell-cycle distribution was assessed by PI staining followed by flow cytometric analysis. Following treatment, cells were harvested by trypsinization, washed twice with phosphate-buffered saline (PBS), and fixed overnight in ice-cold 70% ethanol at −20 °C. After fixation, cells were washed with PBS to remove residual ethanol and incubated with a staining solution containing 50 µg/mL PI and 100 µg/mL RNase A (Sigma-Aldrich, Merck KGaA, Darmstadt, Germany) for 30 min at 37 °C in the dark.

Cellular DNA content was analyzed using a BD FACSCanto II flow cytometer (BD Biosciences, San Jose, CA, USA), with a minimum of 20,000 events acquired per sample. Debris and cell doublets were excluded during the gating procedure to ensure analysis of single-cell populations. The proportions of cells in the G0/G1, S, and G2/M phases were quantified using ModFit LT software(version 5.0; Verity Software House, Topsham, ME, USA). Experiments were performed in three independent biological replicates.

### 4.10. Flow Cytometric Evaluation of ΔΨm

Changes in ΔΨm were evaluated using the cationic fluorescent probe JC-1 (5,5′,6,6′-tetrachloro-1,1′,3,3′-tetraethylbenzimidazolylcarbocyanine iodide; Cayman Chemical, Ann Arbor, MI, USA). HeLa cells were seeded into 6-well plates and allowed to attach for 24 h under standard culture conditions. Cells were subsequently treated with AsA, CDDP, or the AsA + CDDP combination at their respective 48 h IC_50_-based concentrations for 48 h. Untreated cells served as the control group.

Following treatment, both adherent and floating cells were collected, washed twice with phosphate-buffered saline (PBS), and incubated with 2 µM JC-1 working solution for 30 min at 37 °C under light-protected conditions. After staining, cells were washed with PBS to remove excess dye and immediately subjected to flow cytometric analysis.

Fluorescence acquisition was performed using a BD FACSCanto II flow cytometer (BD Biosciences, San Jose, CA, USA), and a minimum of 10,000 events were acquired for each sample under identical instrument settings. Cellular debris was excluded according to forward scatter (FSC) and side scatter (SSC) characteristics, followed by doublet discrimination using FSC-A/FSC-H gating. JC-1 monomers, indicative of mitochondrial membrane depolarization, were detected in the FITC channel as green fluorescence, whereas JC-1 aggregates, representing polarized mitochondria with preserved membrane potential, were detected in the PE channel as red fluorescence [37].

Flow cytometric data were analyzed using FlowJo software (version 10.0; BD Life Sciences, Ashland, OR, USA). ΔΨm was expressed as the ratio of JC-1 red-to-green fluorescence intensity (PE/FITC). A decrease in the red/green fluorescence ratio was interpreted as mitochondrial membrane depolarization and loss of ΔΨm. All experiments were performed in three independent biological replicates.

### 4.11. Colorimetric Assessment of Caspase-3/7 Activity

Caspase-3/7 activity was determined using a colorimetric assay based on the cleavage of the caspase-3/7-specific substrate Ac-DEVD-p-nitroanilide (Ac-DEVD-pNA). Following treatment, cells from the control, AsA, CDDP, and AsA + CDDP groups were collected, washed twice with ice-cold phosphate-buffered saline (PBS), and lysed in cell lysis buffer. Cell lysates were incubated on ice and subsequently centrifuged to remove cellular debris. The resulting supernatants were collected, and equal amounts of protein were used for the enzymatic assay.

Cell lysates were incubated with reaction buffer containing dithiothreitol (DTT) and Ac-DEVD-pNA substrate in a 96-well microplate at 37 °C for 1 h. Cleavage of Ac-DEVD-pNA by activated caspase-3/7 resulted in the release of the chromophore p-nitroaniline (pNA), which was quantified by measuring absorbance at 405 nm using a microplate reader.

Background absorbance was subtracted from each measurement, and caspase-3/7 activity was normalized to the corresponding protein content. Results were expressed as fold change relative to the untreated control group, which was defined as 1.0. All measurements were performed in three independent biological experiments.

### 4.12. Assessment of Oxidative Stress and Antioxidant Defense

Intracellular ROS generation was assessed using 2′,7′-dichlorodihydrofluorescein diacetate (DCFH-DA; Sigma-Aldrich, Merck KGaA, Darmstadt, Germany). Following treatment, cells were washed with phosphate-buffered saline (PBS) and incubated with 10 µM DCFH-DA for 30 min at 37 °C in the dark. After incubation, cells were washed to remove excess probe, and DCF fluorescence intensity was measured using a fluorescence microplate reader. ROS levels were normalized to the untreated control group and expressed as fold change relative to control.

Lipid peroxidation was evaluated by measuring malondialdehyde (MDA) levels using an MDA Assay Kit (Elabscience Biotechnology Inc., Houston, TX, USA) according to the manufacturer’s instructions. Following treatment, cells were collected, lysed, and centrifuged to remove cellular debris. The resulting supernatants were used for biochemical analyses. MDA levels were normalized to total protein content and expressed as nmol/mg protein.

Cellular antioxidant status was further evaluated by measuring SOD activity and reduced glutathione (GSH) levels using commercially available SOD and GSH Assay Kits (Elabscience Biotechnology Inc., Houston, TX, USA), respectively, according to the manufacturer’s protocols. SOD activity and GSH levels were normalized to total protein content and expressed using the units specified for the respective assays. All measurements were performed in three independent biological experiments.

### 4.13. ROS Analysis and NAC-Mediated Rescue

To investigate the contribution of oxidative stress to AsA + CDDP-induced cytotoxicity, intracellular ROS generation and NAC-mediated rescue were evaluated [35]. For ROS analysis, cells were assigned to five groups: control, AsA, CDDP, AsA + CDDP, and NAC + AsA + CDDP. NAC (Sigma-Aldrich, Merck KGaA, Darmstadt, Germany) was freshly prepared in sterile culture medium and used at a final concentration of 5 mM. Cells in the NAC + AsA + CDDP group were pretreated with NAC for 1 h before exposure to the combination. AsA and CDDP were subsequently administered at their respective 48-h IC_50_-based concentrations, and cells were incubated for 48 h.

Intracellular ROS generation was assessed using 2′,7′-dichlorodihydrofluorescein diacetate (DCFH-DA; Sigma-Aldrich, Merck KGaA, Darmstadt, Germany). At the end of the treatment period, cells were washed with phosphate-buffered saline (PBS) and incubated with 10 µM DCFH-DA for 30 min at 37 °C in the dark. Excess probe was removed by washing with PBS, and DCF fluorescence was measured using a fluorescence microplate reader. Fluorescence values were background-corrected, normalized to the untreated control group, and expressed as fold changes in intracellular ROS levels.

To assess whether NAC pretreatment attenuated combination-induced cytotoxicity, cell viability was measured in the control, AsA + CDDP, and NAC + AsA + CDDP groups using the XTT assay under the same treatment conditions. Viability was expressed as a percentage of the untreated control. NAC-mediated rescue was assessed by comparing viability in the AsA + CDDP and NAC + AsA + CDDP groups. All experiments were performed in three independent biological replicates.

#### 4.13.1. Three-Dimensional Tumor Spheroid Formation and Treatment

A 3D tumor spheroid model was established using HeLa cells to evaluate the effects of AsA, CDDP, and their combination under conditions partially reproducing the structural organization, diffusion limitations, and cell–cell interactions characteristic of solid tumors [45].

HeLa cells in the logarithmic growth phase were seeded into ultra-low attachment round-bottom 96-well plates (Corning Inc., Corning, NY, USA) at a density of 3 × 10^3^ cells/well in a final volume of 200 µL complete DMEM. Plates were incubated at 37 °C in a humidified atmosphere containing 5% CO_2_ to allow spontaneous spheroid aggregation and maturation. Compact and morphologically uniform spheroids formed within 48–72 h and were selected for subsequent experiments.

Following spheroid formation, the culture medium was carefully replaced with fresh medium containing AsA, CDDP, or the AsA + CDDP combination. Treatment concentrations were selected based on the IC_50_ values obtained from the 2D monolayer experiments and were used as operational reference concentrations for the 3D experiments. Considering the diffusion limitations and reduced proliferation kinetics associated with multicellular spheroids, treatment exposure was maintained for 72 h. Untreated spheroids were used as controls. Unless otherwise specified, all spheroid experiments were performed in three independent biological replicates.

#### 4.13.2. Bright-Field Imaging and Morphological Evaluation of Spheroids

Treatment-associated morphological alterations in HeLa spheroids were evaluated using an inverted bright-field microscope (Olympus CKX53, Olympus Corporation, Tokyo, Japan). Representative images were acquired using identical optical and exposure settings across all experimental groups to ensure comparability. Spheroid morphology was qualitatively evaluated according to overall compactness, structural integrity, border regularity, and the presence of cellular dispersion or fragmentation.

#### 4.13.3. Quantitative Analysis of Spheroid Diameter

Spheroid size was quantitatively evaluated using ImageJ software (version 1.53; National Institutes of Health, Bethesda, MD, USA). For each spheroid, the diameter was calculated as the mean of two perpendicular measurements obtained across the widest regions of the spheroid. Measurements were obtained from spheroids generated in three independent biological experiments, and mean spheroid diameter values were expressed in micrometers (µm).

#### 4.13.4. ATP-Based Viability Analysis of 3D Tumor Spheroids

Cell viability within HeLa spheroids was determined using the CellTiter-Glo^®^ 3D Cell Viability Assay (Promega, Madison, WI, USA) according to the manufacturer’s instructions. Following the 72 h treatment period, an equal volume of CellTiter-Glo^®^ 3D reagent was added directly to each well to facilitate complete spheroid disruption and ATP release. After incubation to allow stabilization of the luminescent signal, luminescence intensity was measured using a microplate reader (BioTek Instruments, Winooski, VT, USA). Viability values were normalized to untreated control spheroids and expressed as percentages relative to the control group.

#### 4.13.5. Live/Dead Fluorescence Imaging and Quantification of Spheroid Cytotoxicity

Treatment-associated cytotoxicity within HeLa spheroids was further evaluated using fluorescence-based live/dead staining with Calcein-AM and Ethidium homodimer-1 (Thermo Fisher Scientific, Waltham, MA, USA).

Following treatment and staining, spheroids were visualized using an inverted fluorescence microscope (Olympus CKX53, Olympus Corporation, Tokyo, Japan) equipped with appropriate excitation and emission filter sets. Images were acquired using identical focal planes, exposure parameters, and acquisition settings across all experimental groups without post-acquisition image enhancement or manipulation.

Green Calcein-AM fluorescence and red EthD-1 fluorescence were quantified using ImageJ software (version 1.53; National Institutes of Health). Relative green and red fluorescence-signal fractions were calculated from the fluorescence-intensity measurements obtained in three independent biological experiments. These fluorescence-derived fractions represent relative signal distributions and do not constitute direct counts of viable and non-viable cells. The fluorescence patterns were interpreted together with bright-field morphology and ATP-based viability measurements to assess treatment-associated changes in the 3D spheroids.

### 4.14. Total RNA Extraction and Reverse Transcription

Total RNA was isolated from control and treated cells using TRIzol Reagent (Invitrogen, Thermo Fisher Scientific, Waltham, MA, USA) according to the manufacturer’s instructions. RNA concentration and purity were assessed using a NanoDrop One spectrophotometer (Thermo Fisher Scientific, Waltham, MA, USA). RNA samples with an A260/A280 ratio of approximately 1.8–2.0 were considered suitable for downstream analysis.

Complementary DNA (cDNA) was synthesized from 1 µg of total RNA using the High-Capacity cDNA Reverse Transcription Kit (Applied Biosystems, Thermo Fisher Scientific, Waltham, MA, USA) according to the manufacturer’s protocol. The resulting cDNA was used as the template for subsequent quantitative real-time PCR (RT-qPCR) analysis.

### 4.15. RT-qPCR Analysis of Apoptosis- and Proliferation-Related Gene Expression

Quantitative real-time PCR (RT-qPCR) was performed using a QuantStudio 5 Real-Time PCR System (Applied Biosystems, Thermo Fisher Scientific, Waltham, MA, USA) with PowerUp SYBR Green Master Mix (Applied Biosystems, Thermo Fisher Scientific, Waltham, MA, USA).

The expression levels of apoptosis-related genes (*BAX*, *BCL2*, *CASP3*, and *TP53*) and proliferation-related genes (*PCNA*, *CCND1*, and *MKI67*) were analyzed. *GAPDH* was used as the reference gene for normalization. The forward and reverse primer sequences used for RT-qPCR analysis are listed in Table 1. Relative gene expression was calculated using the 2^−ΔΔCt^ method, with the untreated control group serving as the calibrator. Each sample was analyzed in technical triplicate, and gene-expression analyses were performed using three independent biological replicates.

### 4.16. Protein–Protein Interaction Network and Functional Enrichment Analysis

Bioinformatics analyses were performed to characterize the molecular interaction network and biological pathways associated with proteins relevant to the experimentally investigated cytotoxic, apoptotic, proliferative, and oxidative stress responses. Protein–protein interaction (PPI) analysis was conducted using the STRING database (version 12.0; https://string-db.org/; accessed on 15 September 2026). The network included BAX, BCL2, CASP3, TP53, AKT1, MAPK1, MAPK8, EGFR, NFKB1, and JUN, which were selected based on their established involvement in apoptosis, cell proliferation, survival signaling, and oxidative stress-related pathways.

The resulting PPI network was imported into Cytoscape software (version 3.10.3) for network visualization and topological analysis. Functional enrichment analysis was performed using Gene Ontology (GO) Biological Process terms and Kyoto Encyclopedia of Genes and Genomes (KEGG) pathways to identify biological processes and signaling pathways represented within the protein network. Enrichment results were interpreted in the context of the experimentally observed changes in cytotoxicity, apoptosis, cell proliferation, and oxidative stress.

### 4.17. Statistical Analysis and Data Processing

All experiments were performed using three independent biological replicates unless otherwise specified. Technical replicates within each biological experiment were averaged before statistical analysis so that the biological replicate, rather than the individual technical measurement, represented the experimental unit. Quantitative data are presented as mean ± standard deviation (SD). Statistical analyses and graphical representations were performed using GraphPad Prism version 10.0 (GraphPad Software, Boston, MA, USA).

For experiments involving a single independent factor with more than two groups, including apoptosis, caspase-3/7 activity, ΔΨm, oxidative stress parameters, gene-expression analyses, and 3D spheroid endpoints, differences among groups were evaluated using one-way analysis of variance (ANOVA). When a significant overall effect was detected, Tukey’s multiple-comparisons test was used for pairwise comparisons among experimental groups.

For experiments involving two independent factors, including concentration- and time-dependent cell-viability experiments, two-way ANOVA was performed to evaluate the main effects of each factor and their interaction. Where appropriate, multiple comparisons were subsequently performed with correction for multiplicity.

Dose–response curves for AsA and CDDP were generated by nonlinear regression using a variable-slope dose–response model, and IC_50_ values were estimated from the fitted curves with corresponding 95% confidence intervals. Drug-interaction analysis was performed separately using the Chou–Talalay method, as described above.

For NAC rescue experiments, differences among the control, NAC, AsA + CDDP, and NAC + AsA + CDDP groups were evaluated using one-way ANOVA followed by Tukey’s multiple-comparisons test. This analysis was applied independently to intracellular ROS and cell-viability measurements to determine whether NAC significantly attenuated the effects induced by the AsA + CDDP combination.

For RT-qPCR analyses, relative gene-expression values calculated using the 2^−ΔΔCt^ method were summarized as fold changes relative to the untreated control group. Statistical comparisons were performed using the corresponding ΔCt values rather than directly testing fold-change values.

For the 3D spheroid experiments, spheroid diameter, ATP-based viability, and relative Calcein-AM/EthD-1 fluorescence-signal fractions were compared across treatment groups using one-way ANOVA followed by Tukey’s multiple-comparisons test. Data are presented as mean ± SD from three independent biological experiments (*n* = 3). All statistical tests were two-sided, and * *p* < 0.05 was considered statistically significant. Where applicable, significance levels were denoted as *p* < 0.05 (*), *p* < 0.01 (**), and *p* < 0.001 (***).

## 5. Conclusions

In conclusion, the present study demonstrates that the AsA + CDDP combination exerts greater cytotoxic, antiproliferative, and pro-apoptotic effects in HeLa cervical cancer cells than either single-agent treatment, while exhibiting a nearly additive interaction in HaCaT cells. The combination markedly increased intracellular ROS accumulation and oxidative stress, and the partial reversal of ROS elevation and cytotoxicity by NAC supports the involvement of redox-dependent mechanisms in the treatment response. These effects were accompanied by mitochondrial membrane depolarization, an increased *BAX/BCL2* ratio, enhanced *CASP3* expression and caspase-3/7 activity, G2/M-phase accumulation, and suppression of proliferation-associated genes. Bioinformatics analyses further supported the involvement of apoptosis-, p53-, PI3K–AKT-, MAPK-, and cell cycle-related signaling networks. Importantly, the enhanced antitumor response was retained in 3D HeLa spheroids, where combination treatment produced pronounced disruption of spheroid growth, integrity, and viability. Collectively, these findings indicate that the enhanced activity of AsA + CDDP is associated with coordinated oxidative stress, mitochondrial dysfunction, apoptotic signaling, and proliferative suppression across both 2D and 3D cervical cancer models. Further studies incorporating additional cervical cancer and non-malignant cervical cell models, protein-level validation, and in vivo systems are warranted to determine the translational relevance and therapeutic potential of this combination.

## Figures and Tables

**Table 1:** Primer sequences used for RT-qPCR analysis.

Gene	Forward Primer (5′ → 3′)	Reverse Primer (5′ → 3′)
*BAX*	CCCGAGAGGTCTTTTTCCGAG	CCAGCCCATGATGGTTCTGAT
*BCL2*	GGTGGGGTCATGTGTGTGG	CGGTTCAGGTACTCAGTCATCC
*CASP3*	CATGGAAGCGAATCAATGGACT	CTGTACCAGACCGAGATGTCA
*TP53*	CAGCACATGACGGAGGTTGT	TCATCCAAATACTCCACACGC
*PCNA*	CCTGCTGGGATATTAGCTCCA	CAGCGGTAGGTGTCGAAGC
*CCND1*	GCTGCGAAGTGGAAACCATC	CCTCCTTCTGCACACATTTGAA
*MKI67*	ACGCCTGGTTACTATCAAAAGG	CAGACCCATTTACTTGTGTTGGA
*GAPDH*	GGAGCGAGATCCCTCCAAAAT	GGCTGTTGTCATACTTCTCATGG

## Data Availability

The original contributions presented in this study are included in the article. Further inquiries can be directed to the corresponding authors.

## Abbreviations

2D	two-dimensional
3D	three-dimensional
AsA	ascorbic acid
ATCC	American Type Culture Collection
CDDP	cisplatin
CI	combination index
DCFH-DA	2′,7′-dichlorodihydrofluorescein diacetate
DMEM	Dulbecco’s Modified Eagle Medium
EDTA	ethylenediaminetetraacetic acid
EGFR	epidermal growth factor receptor
FBS	fetal bovine serum
FITC	fluorescein isothiocyanate
GSH	glutathione
HPV	human papillomavirus
IC_50_	half-maximal inhibitory concentration
JC-1	5,5′,6,6′-tetrachloro-1,1′,3,3′-tetraethylbenzimidazolylcarbocyanine iodide
MDA	malondialdehyde
NAC	N-acetylcysteine
NFKB1	nuclear factor kappa B subunit 1
PI	propidium iodide
PPI	protein–protein interaction
ROS	reactive oxygen species
RT-qPCR	reverse transcription quantitative polymerase chain reaction
SOD	superoxide dismutase
TP53	tumor protein p53
XTT	2,3-bis-(2-methoxy-4-nitro-5-sulfophenyl)-2H-tetrazolium-5-carboxanilide
ΔΨm	mitochondrial membrane potential
